# Cost-effectiveness of a combination strategy to enhance the HIV care continuum in Swaziland: Link4Health

**DOI:** 10.1371/journal.pone.0204245

**Published:** 2018-09-17

**Authors:** Elizabeth R. Stevens, Lingfeng Li, Kimberly A. Nucifora, Qinlian Zhou, Margaret L. McNairy, Averie Gachuhi, Matthew R. Lamb, Harriet Nuwagaba-Biribonwoha, Ruben Sahabo, Velephi Okello, Wafaa M. El-Sadr, R. Scott Braithwaite

**Affiliations:** 1 Department of Population Health, NYU School of Medicine, New York, NY, United States of America; 2 ICAP at Columbia University, New York, NY, United States of America; 3 Department of Epidemiology, Mailman School of Public Health, Columbia University, New York, NY, United States of America; 4 ICAP at Columbia University, Mbabane, Swaziland; Western University, CANADA

## Abstract

**Introduction:**

Link4Health, a cluster-RCT, demonstrated the effectiveness of a combination strategy targeting barriers at various HIV continuum steps on linkage to and retention in care; showing effectiveness in achieving linkage to HIV care within 1 month plus retention in care at 12 months after HIV testing for people living with HIV (RR 1.48, 95% CI 1.19–1.96, p = 0.002). In addition to standard of care, Link4Health included: 1) Point-of-care CD4+ count testing; 2) Accelerated ART initiation; 3) Mobile phone appointment reminders; 4) Care and prevention package including commodities and informational materials; and 5) Non-cash financial incentive. Our objective was to evaluate the cost-effectiveness of a scale-up of the Link4Health strategy in Swaziland.

**Methods and findings:**

We incorporated the effects and costs of the Link4Health strategy into a computer simulation of the HIV epidemic in Swaziland, comparing a scenario where the strategy was scaled up to a scenario with no implementation. The simulation combined a deterministic compartmental model of HIV transmission with a stochastic microsimulation of HIV progression calibrated to Swaziland epidemiological data. It incorporated downstream health costs potentially saved and infections potentially prevented by improved linkage and treatment adherence. We assessed the incremental cost-effectiveness ratio of Link4Health compared to standard care from a health sector perspective reported in US$2015, a time horizon of 20 years, and a discount rate of 3% in accordance with WHO guidelines.[1] Our results suggest that scale-up of the Link4Health strategy would reduce new HIV infections over 20 years by 11,059 infections, a 7% reduction from the projected 169,019 cases and prevent 5,313 deaths, an 11% reduction from the projected 49,582 deaths. Link4Health resulted in an incremental cost per infection prevented of $13,310 and an incremental cost per QALY gained of $3,560/QALY from the health sector perspective.

**Conclusions:**

Using a threshold of <3 x per capita GDP, the Link4Health strategy is likely to be a cost-effective strategy for responding to the HIV epidemic in Swaziland.

## Introduction

Swaziland has the highest prevalence of HIV in the world. With a population of 1.1 million persons, Swaziland has an estimated adult (age 18–49 years) HIV prevalence of 31% and an estimated HIV incidence of 2.4%.[2–4] The country has made impressive strides to respond to the epidemic by increasing the number of HIV-positive adults on antiretroviral therapy (ART) from 72,402 in 2011 to 147,274 in 2015.[5] However, evidence shows that linkage to and retention in care after ART initiation remains suboptimal.[6, 7]

In order to garner the individual and societal benefits from ART, HIV-positive individuals must achieve all steps of the HIV care continuum.[8–11] The care continuum has been shown to be fragile with critical gaps reported from multiple studies conducted in sub-Saharan African countries including high attrition from testing to linkage to care and suboptimal retention in ongoing care.[12–20] Despite the interdependent nature of the steps in the HIV care continuum, most studies have only considered the effect of an individual intervention on one step in the HIV care continuum,[21–24] and few have studied a combination approach that targets multiple barriers along the continuum.[25]

The Link4Health Study, a cluster-randomized controlled trial that evaluated a combined multi-intervention strategy aimed at various steps in the HIV care continuum demonstrated significant improvement in the primary outcome of linkage to HIV care within 1 month of HIV testing *plus* retention in care at 12 months after HIV testing for people living with HIV (PLWH) in Swaziland (RR 1.48, 95% CI 1.19–1.96, p = 0.002).[26] The study compared standard of care alone with standard of care along with multiple evidence-based structural, biomedical, and behavioral interventions, including point of care CD4+ cell count testing, rapid ART initiation, SMS visit reminders, financial incentives, and provision of health packages to motivate linkage and retention. The study design was described previously.[26, 27]

Neither, the cost-effectiveness of the Link4health in Swaziland and similar contexts, nor other similar multi-component interventions, however, have been assessed. We aimed to evaluate the cost-effectiveness of this scale-up through use of a computer simulation of HIV progression and transmission, comparing a scenario in which the Link4Health strategy was scaled up with a counterfactual scenario with no implementation of the Link4Health strategy.

## Methods

Analyses were performed using a combined stochastic natural history model and deterministic, compartment model of the HIV epidemic in Swaziland. A previously developed model of HIV progression and transmission for east Africa [28] was modified to be calibrated to Swaziland epidemiological data and parameterized with the observed effects and costs of the Link4Health strategy (Table 1). Using the simulation, the impact and cost-effectiveness of a nationwide scale-up of the Link4Health strategy in Swaziland was projected.

**Table 1:** Key input parameters.

Description of parameter input	Value	References
**Alcohol use**		
Prevalence of unhealthy alcohol use (Male/Female)	9.7%/2%	[29]
Relative risk of unhealthy alcohol use on condom misuse or non-use	1.29	[30]
Relative risk of unhealthy alcohol use on non-HIV STIs	1.72	[30]
Relative risk of unhealthy alcohol use on non-adherence to ART	2.33	[30]
**Sexual risk behaviors**		
Proportion abstinent (M/F)	5%/10%	[31–34]
Proportion monogamous (M/F)	31%/69%	[34–36]
Proportion in multiple, concurrent relationships (M/F)	56%/17%	Assumption
Proportion who are clients of (men) or who are CSW (female) (M/F)	8%/4%	[37, 38]
Frequency of sex acts (per year)	104	Assumption
Duration of relationship	1y-30y	Assumption
Median number of concurrent partners- non CSW	3	[36]
Median number of concurrent partners- CSW	10	[36]
Probability of consistent condom use	56.8%	[39]
Relative risk of unsafe sex (condom nonuse most or all of the time) if aware of HIV status	0.47	[40]
**HIV epidemiology and transmission**		
Adult HIV prevalence (1997)	20.9%	[41]
Probability of transmission per sex act	0.00011–0.01243	[42, 43]
Prevalence of circumcision among adult men	20%	[39]
Modifier of male circumcision on F→M HIV transmission per sex act	RR 0.6	[44]
Modifier of consistent condom use on HIV transmission per sex act	RR 0.2	[45]
Untreated non-HIV STI prevalence	6%	[46]
Probability of HIV testing	40%	[39]
Probability of linkage to HIV care and treatment	50.3%	[39]
Probability of adherence to ART regimen	84%	[47]
**Intervention effects and costs**		
Modifier of Intervention on Linkage and Retention	RR 1.48	RCT
Outpatient care, per year, no ART (visits, SMS, BCPP, Financial incentives)	$324	RCT
On Site CD4+ test available	$26	RCT
**Utilities**		
Decrease in utility with ART	0.053	[48]
Utility with CD4+ count < 50 cells/mm^3^	0.69	[49, 50]
Utility with CD4+ count between 50 cells/mm^3^ and 199 cells/mm^3^	0.79	[50]
Utility with CD4+ count ≥200 cells/mm^3^	0.83	[50]
**Other Costs (2015 US$)**		
Outpatient care, per year, without ART (non-intervention)	$278	RCT
Hospitalization costs, per year	$464	[51]
First-line ART, monthly	$13	[52]
Second-line ART, monthly	$28	[52]
Viral load test	$35	[53]
CD4+ count test (non-intervention)	$11	RCT

Abbreviations: STI, Sexually transmitted infection; CSW, Commercial sex worker; SMS, text messaging; BCPP, Basic care and prevention package

### Model overview

The simulation is composed of two modules. The first module is a Monte Carlo microsimulation of HIV progression that follows a cohort of HIV-positive individuals and predicts time until ART failure, accumulation of resistance mutations, and patient survival. Individuals progress to AIDS and AIDS-related deaths at varying rates depending on whether they adhere with ART and/or develop resistance to ART, based on viral load suppression and CD4+ cell count trajectory. This progression module provides data to inform the second module, a transmission model. This process is described in detail in the Supplementary Material.

**Fig 1:**
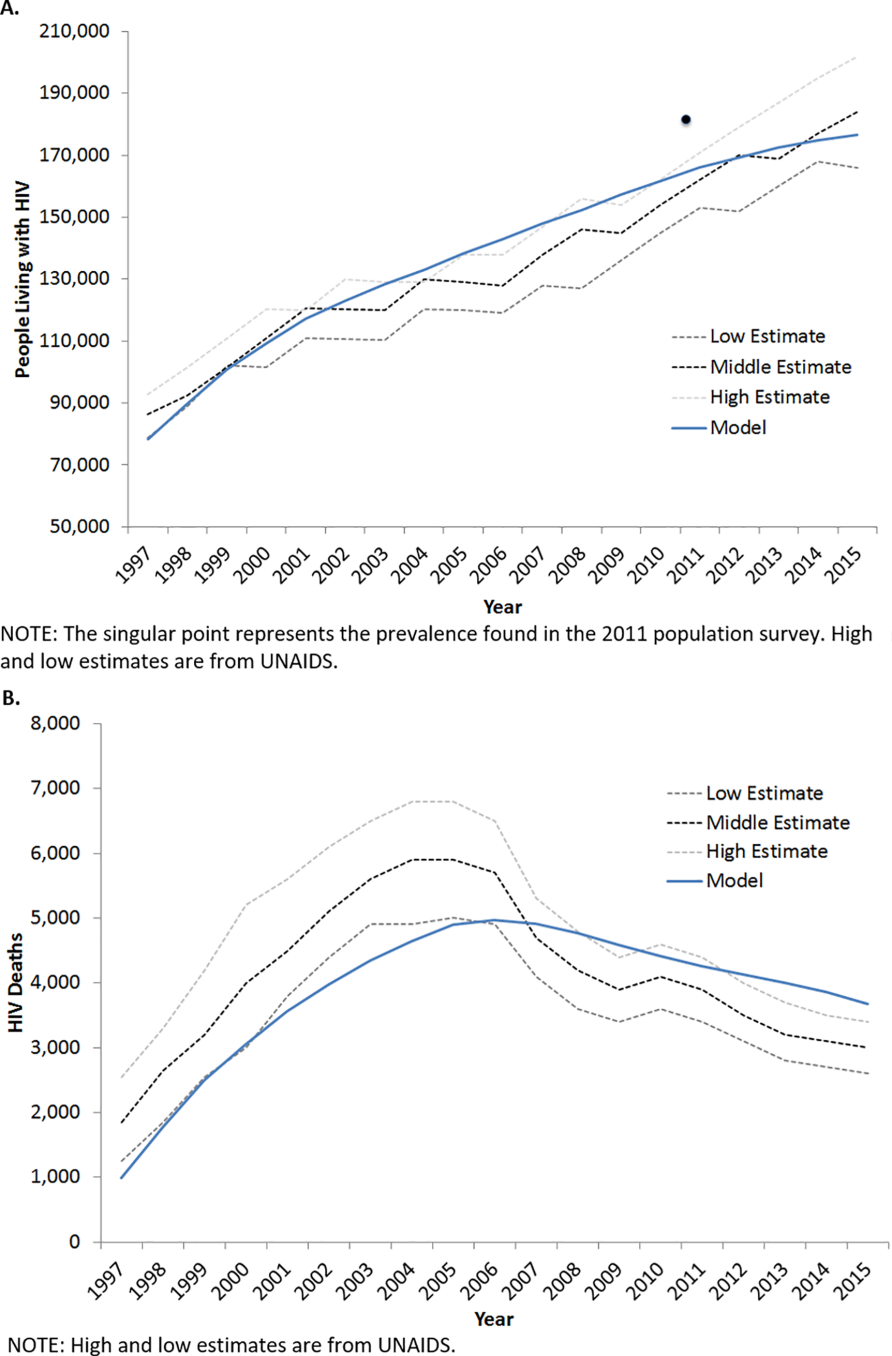
**Calibration (A) Number of persons living with HIV, (B) Number of HIV Deaths.** A. NOTE: The singular point represents the prevalence found in the 2011 population survey. High and low estimates are from UNAIDS. B.NOTE: High and low estimates are from UNAIDS.

In the second module, the transmission of HIV through the Swaziland population is predicted by a compartmental model. In the transmission module segments of a hypothetical population can become HIV infected, have their infection detected, and access treatment, which can modify their infectivity. Segments of this population can also modify their risk of transmission by exhibiting behaviors including ART adherence, multiple sexual partnerships, failing to use condoms, and having STIs. For example, an intervention may lead to improved ART adherence, which lowers viral load and extends life expectancy in the progression module. The lowered viral load then decreases the risk of transmitting HIV in the transmission module. Transmission model compartments are stratified by age, sexual activity level, excessive alcohol use, HIV status, and if infected, viral load, CD4+ cell count, and ART resistance pattern. Model calibration did not prioritize individual parameters, but sought to minimize the least squares across the parameters as a whole. The design of the simulation, as well as its calibration and validation, is described in more detail in the Supplementary Material and elsewhere.[28] We used the calibrated simulation to evaluate the impact and value of the Link4Health strategy in Swaziland. The simulation was calibrated to Swaziland epidemiological data with the goal of replicating trends in Swaziland HIV prevalence, incidence, deaths, and PLWH from 1997 to 2015 (Fig 1).[41, 54, 55] Therefore, model inputs, such as HIV prevalence, reflect 1997 HIV data representing the start of the calibration period. The model inputs and the intervention effects are described in more detail in Table 1.

**Fig 2:**
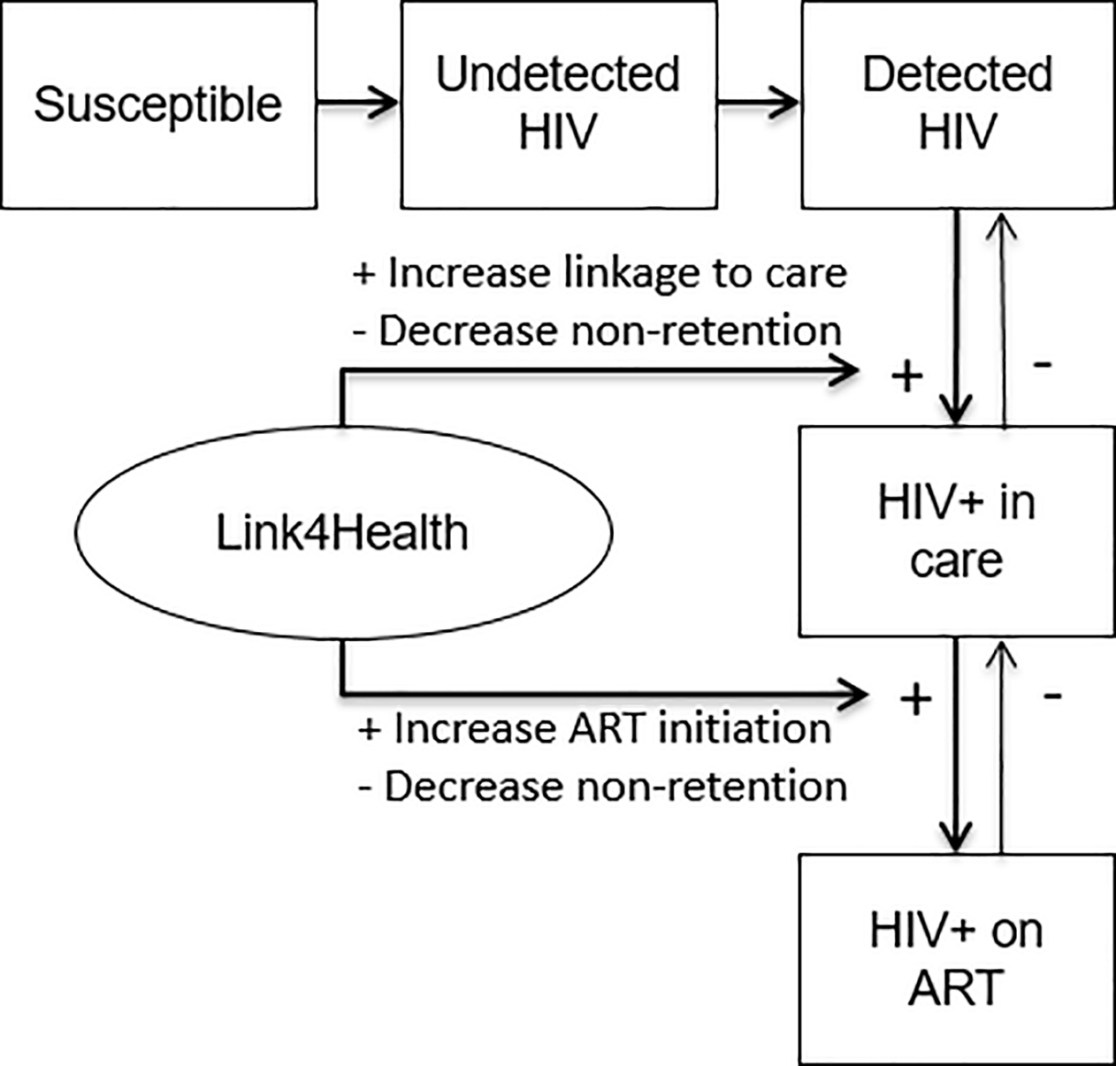
Link4Health impact on model pathways.

The Link4Health strategy was represented in the simulation by a specific set of HIV transmission “pathways” through which it could exert an effect, along with the corresponding target population (e.g. all HIV-positive adults), and an estimated cost per person per year. The effect of the Link4Health strategy (RR 1.48 for linkage to and retention in care) was represented in the transmission model as a multiplier that accelerated pathways for linkage to care and enhanced retention in treatment, in accord with the observed effect size in the study (Fig 2). The effect on linkage and retention was applied to current HIV patients and new HIV diagnoses continuously while the strategy was active. Link4Health also impacts QALYs gained by increasing linkage to and retention in care. The model applied the viral load decrement that is expected with care retention versus non-retention to influence that person’s HIV course (and, therefore, that person’s QALYs), as well as that person’s likelihood of transmitting HIV (and, therefore, other persons’ QALYs). We conservatively assumed that effects only persisted while the strategy was continued.

Outcomes measured included total quality-adjusted life years (QALYs), incremental cost effectiveness ratios (ICERs), number and proportion of new HIV infections prevented and incremental cost per infection averted. Incremental outcomes compared standard of care to standard of care plus the Link4Health intervention. Link4Health cost data was collected prospectively at the time of the trial and represent actual in-trial costs for standard of care and the Link4Health intervention, including the cost of training staff implementing the Link4Health intervention (Table 2). Uncertainty ranges around trial costs were unavailable as all study sites were Ministry of Health (MoH) health facilities with MoH funded staff with defined salaries, and all lab testing were done on a pre-determined schedule based on national guidelines and performed either at the facility or the national laboratory. Consequently, there was little to no variation in treatment costs across study sites. Research-specific costs, such as material development, were not included in the analysis. Non-trial specific costs, including treatment and hospitalization costs were derived from the literature (Table 1). Trial costs were converted from Swazi lilangeni into US dollars and along with cost data collected from the literature were converted into 2015 US dollars using the consumer price index.[56] Costs and effects were discounted at 3%, our time horizon was 20 years, and costs were assessed from a health sector perspective using 2015 US dollars. Other than specifying a finite time horizon, all other aspects of the cost-effectiveness analysis were conducted in line with recommendations by the Panel on Cost-Effectiveness in Health and Medicine.[57] We chose a 20-year rather than infinite time horizon because we found that it was the longest time horizon viewed as credible by stakeholders. A population of 1,098,575 was simulated representing the population of Swaziland at the start of the calibration period in 1997.

**Table 2:** In trial costs per patient and component descriptions.

Item		Step targeted in HIV care continuum	SOC		Link4Health	
			Component Description and Cost Assumptions	Cost per patient	Component Description and Cost Assumptions	Cost per patient
**Point-of-care CD4+ count testing**		Linkage, ART initiation	• Standard CD4+ test after initial HIV diagnosis • Turnaround time approximately 2 weeks • 1 additional clinic visit required to return lab results to patient (15 min staff time of nurse)	**$11.40**	• Point-of-care CD4 assays at the HIV testing site at the time of HIV testing • Turnaround time immediate • Accelerated ART initiation for patients with point-of-care CD4+ count ≤ 350 cells/mm^3^ within 1 week from testing • On site CD4+ test. No staff time for duration of test processing included as routine counseling occurs during processing time; testing supplies; 5 mins for blood draw	**$25.80**
**Outpatient care**				**$278**		**$324**
	Outpatient visit	-	Average cost for standard appointment for ART patients and pre-ART patients, not including treatment	$266.50	Average cost for standard appointment for ART patients and pre-ART patients, not including treatment	$266.50
	Point-of-care CD4+ count testing	Linkage, ART initiation	• Standard CD4+ test after initial HIV diagnosis • Turnaround time approximately 2 weeks • 1 additional clinic visit required to return lab results to patient (15 min staff time of nurse)	$11.40	• Point-of-care CD4 assays at the HIV testing site at the time of HIV testing • Turnaround time immediate • Accelerated ART initiation for patients with point-of-care CD4+ count ≤ 350 cells/mm^3^ within 1 week from testing • On site CD4+ test. No staff time for duration of test processing included as routine counseling occurs during processing time; testing supplies; 5 mins for blood draw	$25.80
	Cell phone visit reminders	Linkage, retention	-	-	• SMS visit reminders 3 days prior to each scheduled visit • SMS reminder within 7 days after a missed visit	$0.90
	Care and Prevention Bags	Retention	-	-	• A health education package every 3 mo. at visits. Included condoms, soap, cotrimoxazole, a pill box, and pictorial education about use of materials and HIV Includes training, materials, staff time	$2.01
	Financial incentives	Linkage, retention	-	-	• Mobile phone credit for those linked to care within 1 month and those completing HIV care clinic visits at 6 and 12 mo. of HIV testing • Mobile phone credit, staff time	$29.14

Abbreviations: ART, antiretroviral therapy; SMS, short message service

### Link4Health intervention

The Link4Health study was a cluster randomized controlled trial performed from 2013 to 2015 in Swaziland that compared standard of care (N = 1,101) to the Link4Health combination strategy plus standard of care (N = 1,096).[26] As noted above, the Link4Health strategy incorporated multiple evidence-based structural, biomedical, and behavioral interventions to improve linkage to and retention in care among adults with HIV in Swaziland. In addition to standard of care, the five interventions combined as package in the Link4Health strategy included: 1) Point-of-care CD4+ count testing. 2) Accelerated ART initiation for eligible participants (CD4+ count ≤ 350 cells/mm^3^ or WHO Stage III/VI) to enable ART initiation within 1 week of diagnosis. 3) Mobile phone appointment reminders. 4) Basic care and prevention package including commodities and informational materials. 5) Non-cash financial incentive for who linked to care within 1 month of HIV testing and those who completed HIV care clinic visits at 6 and 12 months after HIV testing (Table 2). Swazi decision makers were involved at each step of the Link4Health trial and cost-effectiveness analysis and provided guidance to ensure the Link4Health strategy and its analyses were relevant to the Swazi HIV program goals.

### Cost-effectiveness analysis

As a base case analysis we conducted simulations where the Link4Health strategy was activated and calculated the health benefits, costs, and cost-effectiveness ratios of each over the twenty year time horizon. These simulations were compared to a scenario where no additional interventions were implemented. As a sensitivity analysis we varied both intervention efficacy and cost independently across plausible ranges and evaluated their impact on cost-effectiveness. Plausible ranges were determined using trial 95% confidence intervals for effectiveness. It was technically infeasible to do a probabilistic sensitivity analysis as the simulation involves information exchange between a compartmental model and a microsimulation and the run times would be prohibitively long. Between 2013 and 2018 the economic discount rate established by the Central Bank of Swaziland has ranged from 5% to 7.25%.[58] The economic discount rate has been suggested as a surrogate for the social discount rate in developing countries,[59] therefore to encapsulate alternate discount rates that are relevant to Swazi decision making, we also performed sensitivity analyses ranging the discount rate from 5 to 8%. Additionally, in line with evolving ART initiation recommendations, we performed a secondary analysis to simulate the effectiveness of the strategy under the scenario of universal ART adoption, thus eliminating CD4+ testing costs and starting all HIV-positive individuals on ART immediately. We then simulated the Link4Health strategy and calculated the ICERs of the intervention. ICERs measure the additive benefit of each strategy compared with its next best alternative, and interpret this benefit together with its additive cost. A cost per QALY gained value less than three times the per capita GPD (~$9,840) was considered likely to have favorable cost effectiveness, and values equivalent to the per capita GDP ($3,280) were considered favorably cost effective.[60]

## Results

### Base case outcomes

**Fig 3:**
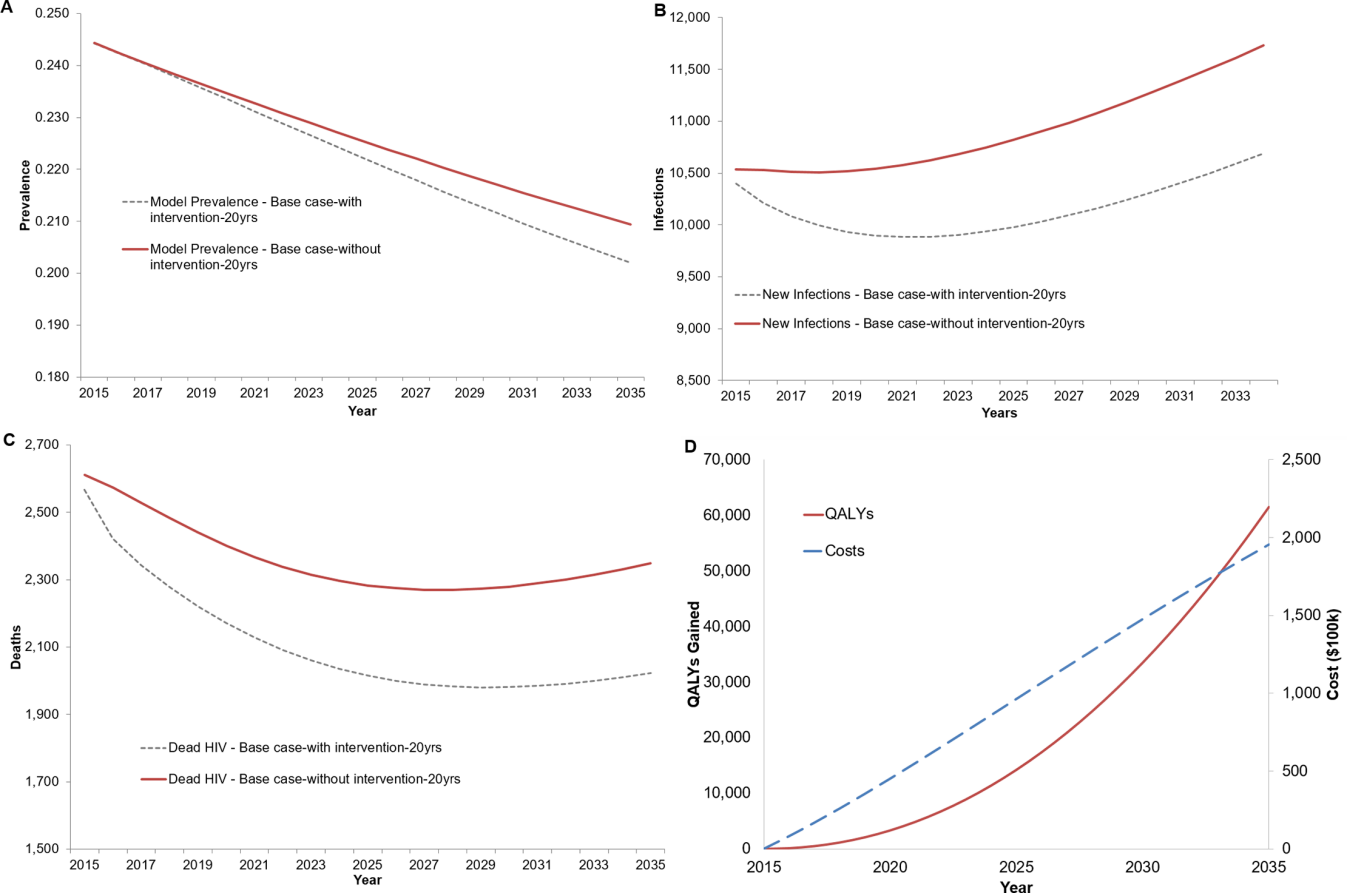
Undiscounted change in (A) HIV Prevalence, (B) Number of new infections, (C) Number of HIV-related Deaths, and (D) Undiscounted cost and undiscounted QALYs gained.

In the base case analysis, the Link4Health strategy reduced the number of new HIV infections over 20 years by 11,059 (6.5%) for total of 157,961 versus 169,019 new infections in Swaziland (Fig 3A) and the HIV prevalence in adults decreased by 0.7% from 20.9% to 20.2% (Fig 3B). The number of HIV-related deaths over 20 years was reduced by 5,313 from 49,582 to 44,270 deaths (Fig 3C).

### Cost effectiveness

When implemented using a health sector perspective, the Link4Health strategy resulted in a total additional cost of $195,700,677 (Fig 2D) and a total discounted additional cost of $147,197,723 over 20 years. Over 20 years the discounted cost per HIV infection prevented was $13,310. Over a 20-year time horizon, the discounted cost per QALY gained was $3,560. Using time horizons of 10 years and 5 years rather than 20 years increased the discounted incremental cost-effectiveness ratio of the Link4Health strategy to $7,195/QALY and $14,122/QALY, respectively, (Table 3) as the costs were mostly borne early whereas the benefits (lengthened life and reduced transmission) mostly occurred later. The discounted cost-effectiveness of Link4Health strategy over 20 years at the highest and lowest range of the 95% confidence interval of intervention effectiveness was $5,572/QALY and $2,911/QALY, respectively.

**Table 3:** Cost-effectiveness by time horizon.

	Total Costs (Discounted)	Total Discounted QALYs	Cost Change	QALYS Gained (Discounted)	ICER ($/QALY)
**20 Yr. Horizon**					
**Standard Care**	$1,263,589,463	19,314,636	-	-	-
**Link4Health**	$1,410,787,186	19,355,978	$147,197,723	38,597	$3,919
**10 Yr. Horizon**					
**Standard Care**	$711,420,043	10,529,352	-	-	-
**Link4Health**	$794,361,248	10,540,879	$82,941,205	10,904	$7,686
**5 Yr. Horizon**					
**Standard Care**	$379,390,695	5,540,162	-	-	-
**Link4Health**	$421,639,679	5,543,153	$42,248,984	2,894	$14,626

### Sensitivity analyses

**Fig 4:**
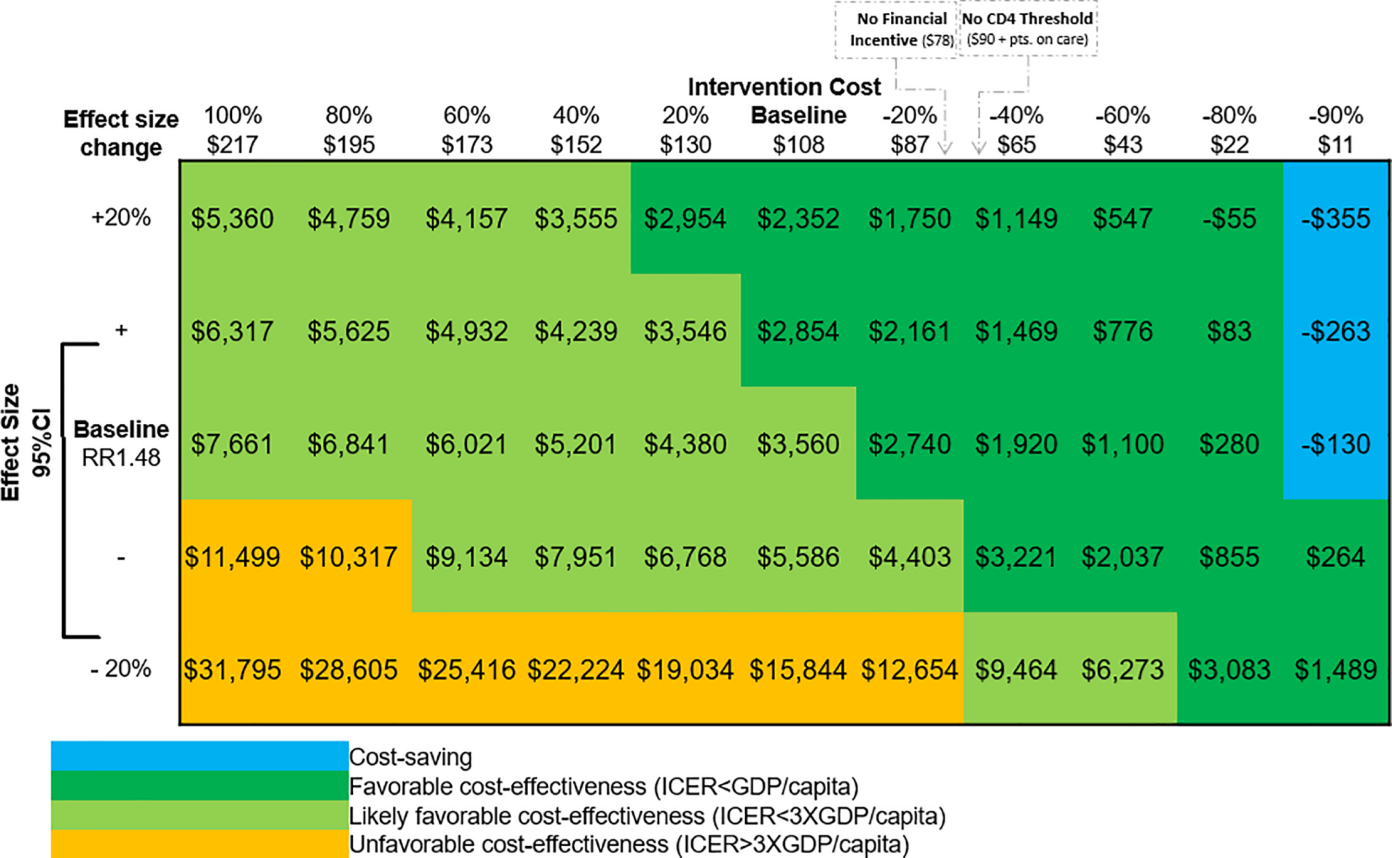
Discounted cost-effectiveness sensitivity analysis.

In one-way and multi-way sensitivity analyses, results regarding costs and benefits were highly stable, with the Link4Health strategy remaining cost effective and improving health across a range of assumptions. In sensitivity analyses to determine the threshold at which Link4Health is no longer cost-effective, reducing the per-patient cost of the Link4Health strategy by 7% (-$8) from baseline ($108) decreased the ICER to make the intervention very cost effective ($/QALY<GPD/Capita) (Fig 4). Reducing the cost by 87% (-$94) made the Link4Health strategy cost-saving (Fig 4). Increasing the strategy cost by 154% (+$166) made the strategy not cost-effective ($/QALY>3xGPD/Capita). The model results were more sensitive to programmatic costs than the cost of care and treatment. Similarly, decreasing strategy effectiveness by 15% decreased new infections averted to 872 infections and HIV deaths to 3,277, decreased discounted QALYs gained to 19,014 QALYs, and made the strategy no longer cost-effective. Increasing the strategy effect size by 4% increased infections averted to 12,603 infections and HIV deaths to 6,055, increased discounted QALYs gained to 44,751 QALYs, and made the strategy very cost-effective (Fig 4). Universal ART decreased the discounted ICER to $2,211/QALY. Increasing the discount rate to 5% and 8% resulted in an ICER of $3,832/QALY and $4,286/QALY, respectively.

## Discussion

We provide projections of the impact and cost-effectiveness of a scale up of a combination strategy of multiple interventions targeting barriers at various HIV continuum steps for linkage to and retention in care among HIV-positive individuals in Swaziland. Few studies have evaluated a combination approach that includes multiple interventions bundled into a coherent strategy that would target numerous barriers along the HIV care continuum,[23, 25, 27, 61] and the cost-effectiveness of such an approach has not previously been evaluated.[62] Our analyses suggest that from a health sector perspective, a scale-up of the Link4Health combination strategy is likely to be cost-effective. Our findings were robust over a range of assumptions regarding cost and effectiveness and the strategy was found to remain cost-effective at shorter time horizons such as 10 years. Examining a range of time horizons demonstrated the potential to underestimate the cost-effectiveness of HIV interventions when only considering shorter horizons due to the frequent longer-term health and cost implications of diseases like HIV/AIDS.

The Link4Health study represents the first study that evaluated multiple interventions packaged in one strategy on both linkage to care and retention in care. Previous studies have found individual elements that increase linkage and retention in care to be cost-effective in other low- and middle- income nations in Africa.[63–65] The cost-effectiveness of the Link4Health strategy falls within the range observed in these single intervention studies, being more cost effective than point of care testing alone ($4,468/DALY),[63] but not quite as cost effective as text messaging alone ($1,024/QALY [adjusted to USD2015]) if DALYs are considered a surrogate for QALYs.[65] This demonstrates the potential benefits of a multicomponent strategy for achieving a greater and more cost-effective impact on the care continuum than implementing standalone interventions. With the potential to further decrease the Link4Health strategy costs, the multicomponent strategy may prove to be the dominant choice for HIV care continuum interventions. A decline in HIV prevalence is projected in Swaziland, even with standard of care, and Link4Health magnifies this decline, which is in large part attributable to the increasing population of Swaziland and low incidence of HIV in new births. Prior to the Link4Health study, there was substantial reluctance to rapidly initiate ART. The Link4Health study, however, has already had a strong influence on the adoption of rapid initiation of ART by the Swazi National AIDs Control Program, thus indicating the interest from stakeholders in incorporating Link4Health-like strategies into the Swazi HIV program. Despite interest in the program and its cost effectiveness as a care continuum intervention, a wide-scale implementation of the Link4health strategy may not be feasible until Swaziland has achieved universal ART coverage at the CD4+ count ≤ 500 cells/mm^3^ threshold. While Swaziland has achieved universal ART coverage under the 2010 WHO guidelines (CD4+ count ≤ 350 cells/mm^3^), it has not achieved universal ART coverage at the CD4+ count ≤ 500 cells/mm^3^ threshold.[66] Therefore, HIV funds may not be available as they are diverted to the more cost effective implementation of universal ART coverage at the CD4+ count ≤ 500 cells/mm^3^ threshold ($245 to $1,746/DALY [adjusted to USD2015]).[67]

The largest portion of program costs was incurred through ART treatment costs, followed by outpatient care, and the cost of CD4+ count testing. Therefore, there is potential to reduce the cost of the HIV treatment program through reductions in the cost of outpatient care and CD4+ count testing as HIV programs shift to differentiated models of care in which stable HIV patients on ART may decrease the number and or frequency of clinic visits and universal ART initiation replacing the need for CD4+ count testing. For example, in some resource-limited settings, patients who achieve viral suppression and are clinically stable receive ongoing HIV care, including medications, in community adherence clubs, which decrease the number of clinic visits.[68, 69] The 2015 Swaziland HIV strategy supports the development of HIV support groups, however universal test and treat, while under consideration, is not part of current HIV guidelines.[66] Further reductions in program costs are likely feasible because, while financial incentives have shown promise in increasing HIV treatment in some interventions,[70] a comparable level of effectiveness to that in the Link4Health study was found in a trial performed in Mozambique of a similar intervention that did not include the financial incentive component.[71] Removing the financial incentives from the Link4Health strategy has the potential to decrease the strategy cost by nearly 30% and result in an even more favorable cost-effectiveness at <1 times the GDP. The adoption of “treat-all” WHO recommendations in Swaziland may eliminate the need for CD4+ count testing, reducing programmatic costs by nearly 25% and improving potential cost-effectiveness to $2,211/QALY (Fig 4).[72]

Our analysis has a number of limitations. First, because of the compartmental nature of the transmission model, the effect on retention in care was represented through improvement of ART adherence and does not account for the other potential benefits gained such as access to other clinical assessments and health counselling. As a result, our analyses may underestimate the total health benefits generated by the Link4Health strategy. Second, the analysis included the same strategy costs for all individuals enrolled in the study, including those that did not ultimately receive the full intervention. This, however, likely overestimates the total cost of implementation as individuals not in HIV care would not incur the full strategy cost. Similarly, we assumed that HIV care visit costs would be the same for patients both on ART and those not on ART. In practice, however, these costs may differ due to factors such as the professional level of the healthcare worker. These differences in visit costs, however, are likely to be negligible when the cost ART treatment is considered. Third, not all model inputs were available for a Swazi-specific setting, which may impact cost-effectiveness. During model validation, the model was permitted to fit some indicators better than others in order to avoid overfitting to a particular data source. The robust cost-effectiveness results across sensitivity analyses, however, indicate that minor variations in these inputs are unlikely to substantially affect the results. Fourth, the Link4Health strategy was carried out in a population that has high prevalence of HIV compared to other countries in the world. Consequently, our results may not be applicable to other populations. Further testing is needed to investigate the Link4Health strategy’s effectiveness in populations outside of high HIV prevalence countries and contexts. Fifth, the nature of the Link4Health trial, which examined the implementation of a package of interventions together and did not isolate the impact of each component separately, made this analysis unable to estimate how each component individually affects the cost-effectiveness of the combined strategy. Similarly, due to the model structure it is not technically feasible to separate how annuals costs will accrue for each relevant budget holder. Finally, it is worth noting that the use of a cost-effectiveness threshold of <3 x per capita GDP, while transparent and easily applied, is insufficient to determine whether an intervention should be implemented. The more relevant cost-effectiveness threshold would be the incremental cost-effectiveness ratio of simultaneously resource constrained interventions, as the health gained from any additional funds allocated towards on Link4Health would likely exceed the health lost from removing funds from other interventions.

## Conclusion

The scale-up of the Link4Health strategy would substantially reduce HIV-related deaths and avert new HIV infections. With a favorable value over a 10 year timeframe or longer, using a threshold of <3 x per capita GDP, the Link4Health strategy could be a cost-effective strategy for confronting the HIV epidemic in Swaziland and other low-income countries with a generalized HIV epidemic. Efforts to strategically reduce strategy costs could lead to Link4Health being a highly cost-effective HIV intervention.

## Supporting information

S1 Technical Appendix (DOCX)
