# Supplementary material for: Cost-effectiveness of a combination strategy to enhance the HIV care continuum in Swaziland: Link4Health
Source: PLoS One. 2018 Sep 17;13(9):e0204245. doi: 10.1371/journal.pone.0204245 (PMC6141095; doi:10.1371/journal.pone.0204245)
Supplement: S1 Technical Appendix — (DOCX) [file pone.0204245.s001.docx]

Supplementary Materials

Methods

**HIV Epidemic Model**

We modified a previously described compartmental, deterministic, SIR-type model to represent heterosexual transmission of HIV within an East African setting. This model incorporates equilibrium results from a previously validated stochastic model of HIV disease progression [1, 2].

For additional information or for a working copy of the C++ code used to conduct the analyses outlined in this manuscript please contact the director of our mathematical modeling team at Kimberly.Nucifora@nyumc.org.

**1.2 Epidemic model population definition**

The simulated population was divided into ten 5-yr age groups (*a*), two genders (*k*), and four sexual activity classes (*l*) [Table S1]. These risk groups were selected to capture differences in HIV prevalence, population size, sexual behaviors and transmission risk. For those persons living with HIV/AIDS their disease was represented with a CD4 count category (*cd*),VL category (*v*), and a HIV resistance category (*m*). In addition, the spectrum of infection and engagement in care was represented in a status category (*y*) [Table S1]. The specific combination of age, gender, sexual activity class may be (where appropriate) referred to using “risk strata” (subscript *p)* throughout the remainder of this document. The combination of CD4, viral load and resistance category may be referred to as “HIV state” (subscript *h*) throughout the remainder of this document.

**Table S1.** Components of population matrix

| **Population parameter** | **Abbreviation/Subscript** |  | **Subgroups** |
| --- | --- | --- | --- |
| Age | *i* | *p* | 0-4, 5-9, 10-14, 15-19, 20-24, 25-29, 30-34, 35-39, 40-45, 45-49 |
| Gender | *k* |  | Women(*k=1*), Men (*k=2*) |
| Sexual activity | *l* |  | Abstinent (*l=1*), Monogamous (*l=2*), Multiple partnerships (*l=3*), CSW or clients of CSW (*l=4)* |
| CD4 count | *cd* | *h* | 0-50, 51-200, 201-350, 351-500, >500 cells/mm^3^ |
| VL | *v* |  | 0-2.5, 2.5-3.5, 3.5-4.5, 4.5-5.5, >5.5 log copies/ml |
| HIV resistance | *m* |  | No resistance (m=0), NNRTI only (m=1), PI only (m=2), NRTI only (m=3), NNRTI and NRTI (m=4), PI and NRTI (m=5), NNRTI and PI (m=6), All 3 classes (m=7) |
| Status | *y* |  | Susceptible (y=1), Acute HIV infection (y=2), Chronic HIV infection (not detected) (y=3), Chronic HIV (detected) (y=4),  Chronic HIV (in care) (y=5), Chronic HIV (on ART) (y=6) |

**1.3 Epidemic model structure and equations**

The compartmental model was created through a system of nonlinear differential equations for each spectrum of care group (referred to as status in Table S1) further subdivided by age (a), CD4 category (cd), VL category (v), HIV resistance (m) and risk group (p). Across the spectrum of care, transition from susceptible (S_r_) to acute HIV infection (I^1^_r_) occurs as a result of new transmission events; transition from (I^1^_r_ ) to undetected, chronic HIV infection (I^2^_r_ ) occurs at a constant rate and represents the natural history of acute HIV infection; transition from I^2^_r_ to chronic HIV, detected (I^3^_r_) occurs as a result of HIV testing and this rate can vary in relation to changes in this probability. Transition from I^3^_r_ to chronic HIV, in care (I^4^_r_) occurs as a result of linkage to care. Finally, transition from I^4^_r_ to HIV, on ART (I^5^_r_) occurs once those in care initiate ART, which is directly related to the assumed ART eligibility criteria. From I^5^_r_ the only transition that can be made is to death. [Figure S2] As discussed below, HIV progression (i.e. transitions between CD4 and VL compartments) and HIV-related mortality was modeled using rates developed from a stochastic state-transition model [1, 2].

**Figure S2.** Schematic diagram of HIV transmission model


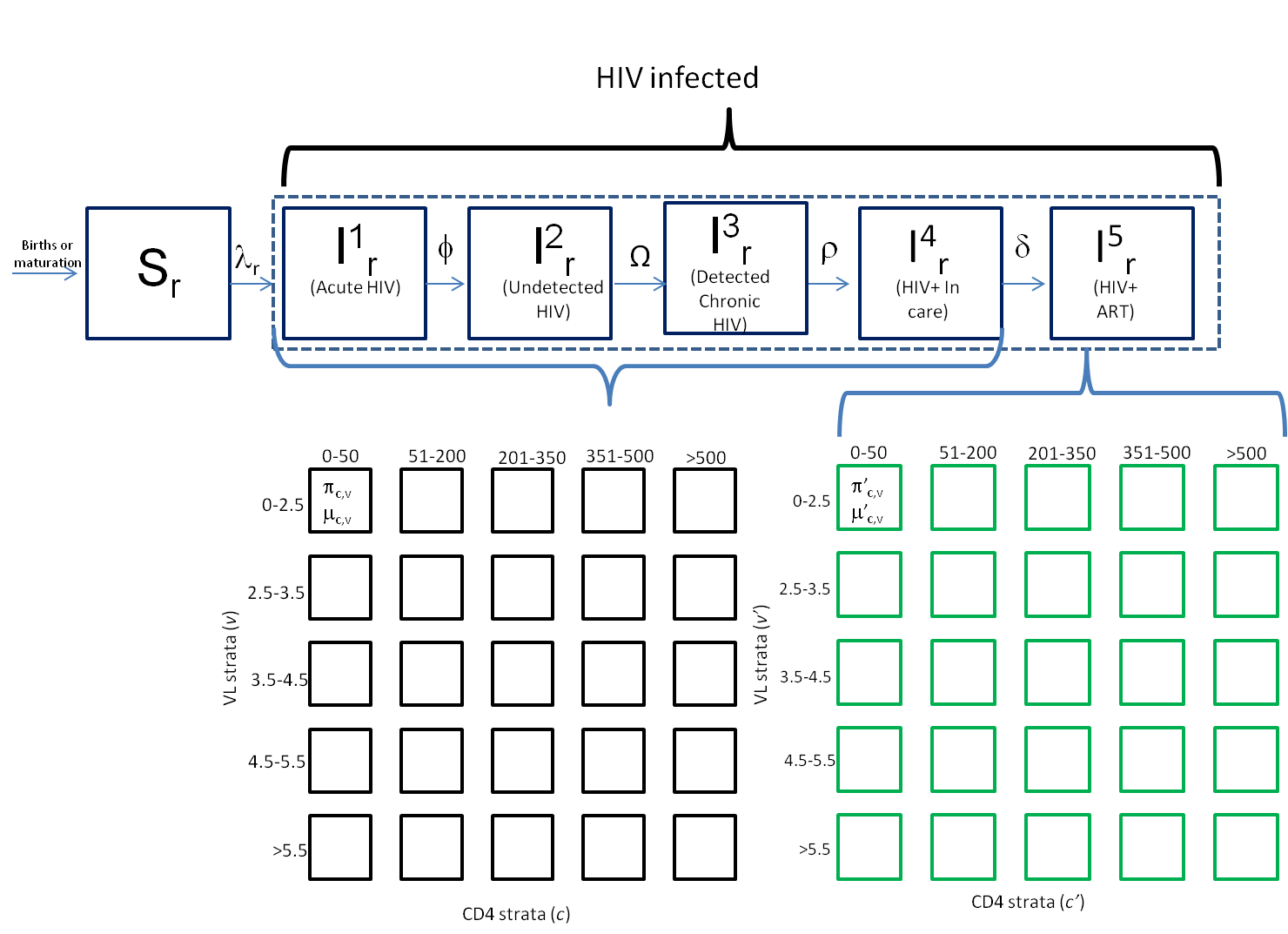


HIV disease progression model

(equilibrium rate tables)

HIV transmission model

**1.3.1 Equations governing transitions**

N = Total population

N’ = HIV infected population

S = N-N’ = Susceptible (HIV-) population

p = risk strata- given combination of age strata (a), gender (k), sexual activity class (l)

h= disease strata – given combination of CD4 strata (cd) , VL strata (vl) and HIV resistance (m) category

μ_h_= HIV-related mortality rate (for a given CD4 strata (cd), VL strata (v), and resistance (m) category)

μ’_h_= HIV-related mortality rate if on cART

μ_age_ = age – related mortality rate

λ = force of infection

γ = birth or maturation

π = rate of transition between any two CD4, VL strata, and resistance categories

φ = flow from acute to chronic HIV infection

η = rate of transition from acute to chronic HIV infection

Ω = rate of HIV testing [= - ln(1-P_test_)/t)]

ρ = rate of linkage to care [= - ln(1-P_link_)/t)]

δ = rate of initiation of ART (once in care)

P_test_= probability/rate of annual HIV testing

P_LTC_= probability/rate of linkage to care (if detected)

P_ART_= probability/rate of initiation of ART (if in care)

1. *d*S_r_/*d*t _=_ γ _r_ – λ^t^_r_ (*t*)S_r_ – μ_age_
2. *d*I^1^_r_/*d*t = λ^t^_r_ (*t*)S_r_ – φ_r_ - μ_age_ - μ*_cd,v_* (where φ_r_ = η*N’_r_)
3. *d*I^2^_r_ / *d*t = φ_r_ - Ω - μ_age_ - μ*_cd,v_*
4. *d*I^3^_r_ / *d*t = Ω – ρ - μ_age_ - μ*_cd,v_*
5. *d*I^4^_r_/*d*t = ρ_r_ – δ_r_ - μ_age_ - μ*_cd,v_* (where δ_r_ = N’_r_*π*_cd<threshold,v_* * PART)
6. *d*I^5^/*d*t = δ_r_ - μ_age_ - μ’*_cd,v_*

**1.4 HIV transmission**

The population is described by two genders (male and female), and four sexual activity groups. Each activity group is defined by a desired relationship duration in years *d* and desired number of concurrent partners *r* . The mathematical framework builds on published work by Garnett & Anderson [3, 4]. Following their structure, the population defined by the model is stratified by gender *k* , age *i* , and sexual activity class *l* . Individuals form partnerships with the opposite gender *k'* from age class *j* and activity class *m*. The notation for duration d_klmij_ would refer to the duration of a partnership formed between someone of sex *k* , activity group *l* and age group *i* with someone of the opposite sex from activity group *m*, age group *j* . In addition to the above parameters, we set a desired frequency of sexual acts (number of acts per year) which applies to both genders and a probability of transmission per sexual act. The following steps were undertaken to create and balance the transmission simulation.

**a) Duration**

When a man and woman form a partnership, we make the assumption that the partner wanting the shorter relationship controls the relationship duration. Thus the duration of the partnership is the minimum of each partner's desired duration.

d_klmij_ = min(d_kli_, d_k'mj_)

**b) Proportional mixing matrix incorporating concurrency**

We begin by calculating the proportionate mixing matrix*.* This indicates the probability that an individual in a given group will partner with an individual from each other group under random, proportionate mixing. Note that instead of using partner change rate in this calculation, we use concurrency to determine the number of partnerships available at a given moment in time.

The proportionate mixing matrix ρ^p^ is defined by:

(1)


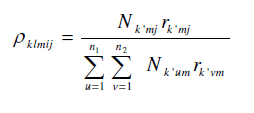


**c) Adding assortativeness of age, activity and age preference to mixing matrix.**

We have incorporated multiple methods (of varying complexity) of specifying degree of assortativeness (the level of preference for like to mix with like) in the model. These methods are described by Garnett and Anderson in their papers **[3, 4]**. In the simplest case, we use one parameter ε to denote the degree of assortative mixing (eq 2).

(2)


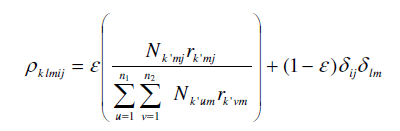


Here, δ_ij_ is the Kronecker delta (i.e. δij = 1 if i = j and 0 otherwise). When ε = 0, mixing is entirely assortative and when ε =1 mixing is proportionate. If we wish to specify degrees of assortativeness for age and activity separately, we use the following equations.


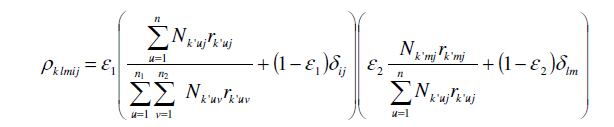
 ( 3)

Here, ε1 specifies the degree of assortative mixing between age classes and E2 specifies the degree of assortative mixing between activity classes. Lastly, we can include a preference for a mismatch between the ages of the sexual partners. The following set of equations builds on the previous where we separately specify assortativeness of age groups, ε1 and assortativeness of activity groups ε2. Additionally, we specify the degree to which members of one sex (generally men) prefer partners of the opposite sex two age groups younger, ε3.

(4)


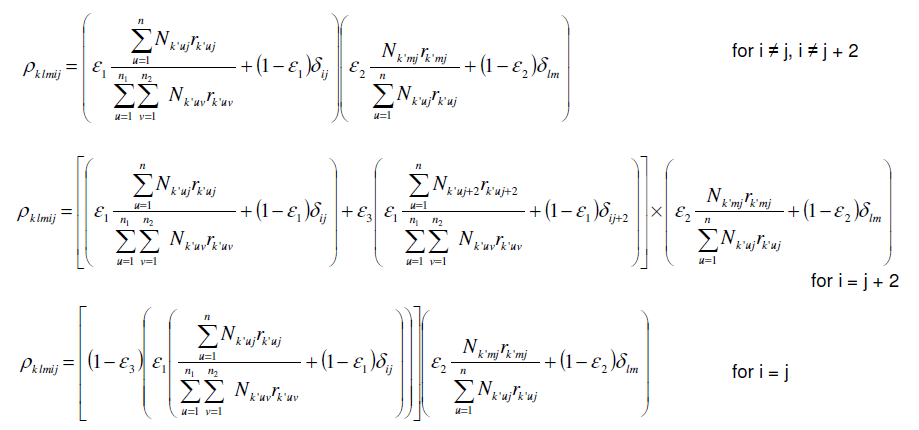


**d) Number of partnerships offered at any moment in time**

The number of partnerships offered by a group kli to a partner group k'mj is the product of the number in that group, the number of concurrent partners for that group, and the probability that the partner will be from the partner group.

numPartnerships_klmij_ = N_klmij_ * r_klmij_ * ρ_klmij_

**e) Compromise on number of partnerships**

When two groups in a partnership are offering each other unequal numbers of partnerships, the lower number of partnerships prevails. Thus one group may get fewer partnerships than it desired.

numPartnershipsCompromise_klmij_ = numPartnershipsCompromise_k'mlji_ = min(numPartnerships_klmij_, numPartnerships_k'mlji_)

**f) Compromise on concurrency**

Now that one group may have had to compromise on the number of partnerships it can obtain with a partner group, we have to revise the concurrency for that group, specific to that partnership. Revisiting our equation from step 4 but using the number of partnerships after compromising,

numPartnershipsCompromise_klmij_ = N_klmij_ * r_klmij_ * ρ_klmi_

Solving for concurrency,

r_klmij_ = numPartnershipsCompromise_klmij_ / (N_klmij_ * ρ_klmi_)

**g) Frequency of sexual acts**

In our model, we assume a overall desired frequency *F* for all individuals. This overall desired frequency is independent of the number of concurrent partners. Thus an individual with only one partner with have sex acts of frequency F with that one partner. An individual with *r* concurrent partners will have frequency F/r with each partner. In step 6, we showed that because one group may have had to compromise their number of partnerships, their

concurrency with a partner group likewise had to be adjusted. We calculate actual frequency fklmij for a group with a specific partner group as

f_klmij_ = F / r_klmij_

**h) Compromise on frequency**

Two individuals who form a partnership must each experience the same frequency within the partnership. If their calculated desired frequencies with each other are unequal, one partner will have to compromise on their desired frequency. In this model, we assume that the lower desired frequency prevails. Thus,

f_klmij_ = f_kmlji_ = min(f_klmij_, f_kmlji_)

**i) Acts per Partnership**

The number of acts within the course of a partnership is equal to the frequency of sex acts multiplied by the duration.

acts_klmij_ = f_klmij_ * d_klmij_

**j) Partner change rate**

The partner change rate c is equal to the number of partnerships per year (in series) multiplied by the number of concurrent partnerships r. The number of partnerships in series is the inverse of the duration. Thus,

c_klimj_ = 1/d_kli_ * r_klimj_ = r_klimj_/d_kli_

**k) Force of Infection**

The force of infection (λ) is defined by


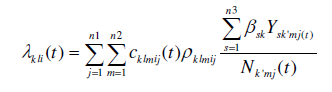
 (5)

where s refers to the infectious states and Ysk'mj(t) is the number of sexual partners of age group j and activity group m in that infectious state. The calculation of Beta is described in equation (7) below. The term Y_sk'mj_(t)/N_k'mj_(t) is then the proportion of sexual partners who are infected.

1.4.1 Calculation of probability of transmission per act and per partnership

We refer to the transmission probability per partnership as β and the transmission probability per sexual act as α. Alpha(α) constants were set based on literature reviews and vary by type of partnership and the infecting partner [Table S4]. These values can be modified by HIV disease status (acute vs. chronic infection), viral load, adherence and specific interventions which act or promote different behavioral or biological modifiers that increase or decrease this probability (e.g. condom use during sex act will decrease the probability of transmission).

Therefore, under different conditions α is calculated using the following equations:

*Infected partner not receiving treatment (and no alpha modifying interventions):*

α_final_= α* α^m^_v_ (6)

where α^m^_v_ is the alpha modifier for a viral load strata *v*

To account for the higher VL (and likely transmission probability resulting) associated with the acute HIV state, if the partner of a susceptible person was in an acute HIV state (I^1^_r_) then the viral load of the infected person was conservatively estimated to be 1.5 log units greater than its set point (and a different VL modifier (α^m^_v_) applied to the above equation [5].

*Infected partner receiving treatment (and no alpha modifying interventions):*

First, an “on treatment” viral load strata for infected population is determined as follows:

VL_treat_ = VL - (VLdec * P_adh_)

and then

VL_treat_ is then assigned a new VL strata (*v’ )* category

α^mt^_v_ is the alpha modifier of the new VL strata on treatment *v’*

The final alpha value is then calculated as in equation (6).

To calculate β we assumed a binomial process where the number of trials referred to the average number of sex acts per partnership (Acts) and the probability of successful transmission is described by α_kKV_ as above. Therefore, beta was calculated using the following equation.

β = 1 -(1 - α_final_)^Acts^ (7)

1. **HIV disease progression**

**2.1 Disease progression model calibration**. We had previously calibrated the disease progression (stochastic, Markov state transition model) module for East Africa by comparing its results with clinical data obtained from the United States Agency for International Development Partnership with the Academic Model Providing Access to Healthcare (USAID-AMPATH) partnership, using a pre-specified battery of comparisons based on our previous calibration methods [1,2]. In particular, our pre-specified calibration criteria involved survival [Figure S2A], time to switching of cART regimen 1 [Figure S2B], time to switching of cART regimen 2 [Figure S2C], and mean CD4 count [Fig S2D]; where “switching” was defined as changing 2 or more cART drugs.

We used statistical methods, previously published by our group, to adjust passively assessed patient mortality by employing random sampling of patients lost to follow-up with active surveillance and estimating overall mortality by combining the data with patients who remained in follow-up. In addition, we estimated the longitudinal trajectories of CD4 counts based on data obtained in the subgroup of USAID-AMPATH patients retained in clinic. Unfortunately, the later data are potentially biased in unpredictable ways (for example, tracking median CD4 count over time might overestimate the true CD4 trajectory as patients with lower CD4 counts may die or be lost-to-follow-up at higher rates than patients with higher CD4 counts). For this reason, we placed the greatest emphasis on ensuring that the survival curves, produced by our simulation model, fit the clinical data well.

Calibration of the HIV transmission module is described in Section 5.

**Figure S2A-D**. Calibration of the stochastic, disease progression model which provided equilibrium results to epidemic simulation. A. survival; B. Time to switch while on 1^st^ ART regimen; C. Time to switch while on 2^nd^ ART regimen; D. Mean change in CD4 count

2A

2B.

2C.

2D.

**2.2 Generation of equilibrium results from disease progression model for use in epidemic, compartmental model**

HIV disease progression was modeled by the inclusion of stationary rates developed from a previously developed and validated computer simulation [1]. {Braithwaite, 2005 #190}Within the epidemic compartmental model CD4^+^ count was categorized into five mutually exclusive compartments **(*cd*)** [0-50, 50-200, 200-350, 350-500, >500 cells/mm^3^. Viral load was categorized into five strata **(*v*)** [0-2.5, 2.5-3.5, 3.5-4.5, 4.5-5.5, >5.5 log copies/ml]. HIV-1 resistance profile was categorized into seven mutually exclusive categories including no resistance , NNRTI class resistance only, PI class resistance only , NRTI class resistance only, both NNRTI and NRTI class resistance, both PI and NRTI class resistance, both NNRTI and PI class resistance, and multidrug resistance (all 3 classes). Upon initialization, compartments representing PLWHA at the start of the simulation were assigned a CD4 count, and VL as represented from the distribution in Table S2. This distribution describing the CD4 and VLs for PLWHA at the start of the simulation were created using published data [6, 7]. We assumed no HIV-1 resistance to anti-retroviral drugs in the population at the start of the simulation (i.e. 1997).

|  |  | CD4 Count Category/Compartment (*c*) | | | | |
| --- | --- | --- | --- | --- | --- | --- |
|  |  | **0-50** | **51-200** | **201-350** | **351-500** | **>500** |
| Viral Load Category or Compartment (*v*) | **0-2.5** | 0.000000 | 0.000000 | 0.003968 | 0.003968 | 0.031746 |
|  | **2.5-3.5** | 0.000000 | 0.000000 | 0.012698 | 0.012698 | 0.073016 |
|  | **3.5-4.5** | 0.005952 | 0.017857 | 0.038095 | 0.046429 | 0.184524 |
|  | **4.5-5.5** | 0.013095 | 0.039286 | 0.061905 | 0.078571 | 0.178572 |
|  | **>5.5** | 0.016667 | 0.050000 | 0.059524 | 0.034524 | 0.036905 |

All persons infected with HIV after the start of the simulation were assigned a CD4 strata of >500 cells/mm^3^ and a viral load from a normal distribution with a mean of 4.45 log copies and standard deviation of 0.78 [8].

**Table S2**. Probability distributions for initialization of CD4 count strata and VL strata within HIV infected compartments

1. Probability distribution (Q*_cd,v_*) of CD4 and VL categories for HIV infected persons at initialization
   - 1. Incorporation of equilibrium results from stochastic disease model into epidemic model

The rate of transition between these compartments ([*cd,v,m*]_(t)_ 🡪 [*cd,v,m*], _(t+1))_; **π**_cd1v1m1cd2v2m2,_ hereafter shortened to **π_h_**) were determined by referencing rate transition tables under two different conditions (on ART and off ART) depending on ART threshold (assumed to be <200 cells/mm^3^ under base case). These rate transition tables were generated from the previously mentioned disease progression model.

This was achieved by using the previously mentioned Braithwaite stochastic HIV progression model to determine the rates of transition between CD4 count, viral load categories, and resistance categories and then substituting these calculated rates into a deterministic model. More specifically, separate one million trial simulations were conducted (under conditions of no ART available and ART available), to generate “off-care” and “on-care” estimates of disease progression. During these simulations state transitions between the aforementioned viral load categories, CD4 categories, and resistance categories were tracked, as well as transitions between any combination of these and HIV-related death. Rates were then calculated and “lookup tables” generated that indexed these state transitions by current CD4, VL, ART status, resistance category and HIV-related death (yes/no).

We evaluated the validity of this construction by subjecting the compartmental model to a similar analysis as that performed in a previous published analysis [2] using the Braithwaite stochastic model after harmonizing the assumptions and inputs between the two models. Results of the comparison between different monitoring strategies were similar between equivalent simulations (stochastic model vs. transmission model). In addition, the rank order of strategies from most effective to least was nearly identical between the two different models. We concluded, therefore, the deterministic model mimics the mean behavior of the stochastic model.

Progression through the spectrum of engagement was modeled as a stepwise dependent process starting from HIV uninfected through HIV infected and on ART [Figure S1]. Initiation of ART was simulated when CD4 category fell beneath stated ART threshold and corresponded to the utilization of the “on care” integrated look-up table. Mortality from HIV (if infected) (μ*_h_*) could occur from any infected compartment while mortality unrelated to HIV (μ_age_) could occur from any compartment. Under conditions of treatment, transitions between CD4, VL strata, resistance strata are referred to as π’_h_ and AIDS related mortality rates as μ’_h_. HIV-related mortality rates (μ_h_ or μ’_h_) were determined from indexing the appropriate HIV progression model lookup tables.

1. **Initialization of population matrix**

We calculated the initial populations of each compartment in the model using Uganda specific epidemiological data for both the HIV negative and HIV positive populations as of 1997 [9-11]. Uganda was chosen as the proxy country for East Africa because at the time of model development the most complete longitudinal epidemiologic data was available for this national jurisdiction. Proportions of HIV infected individuals stratified by age and gender were determined from published epidemiological data [9, 11]. It was assumed that at the start of the simulation (i.e. 1997) that all HIV infected persons were in the undetected or detected compartments but none were in ART treatment compartments. We assumed that at the simulation start time, a HIV prevalence in the adult population of 10.6% and that 15% of infected persons had been tested and were aware of their results [12]. We did not assume any differences in likelihood of HIV testing between genders.

The simulation was run over a 14 year calibration period, representing the HIV epidemic during the period of 1997-2011. During this initialization period from 2003 onwards the baseline (i.e. assumed values for 1997) annual probability of undergoing HIV testing (10%) and the probability of linking to care given HIV positive (0%) were linearly scaled upwards to their assumed 2011 values of 16% and 68% respectively. This was incorporated into the model in order to represent the expansion of ART treatment programs within this setting. At the end of the initialization/calibration period a “snapshot” of the model population was captured and functioned as the initial population for all predictive/analytic simulations described in the manuscript. The initial population after this calibration period is outlined in Table S3. Distributions across sexual activity classes (*l*) can be calculated by multiplying a given cell with its respective probability conditional on gender [Table S3].

**Table S3.** Calculated initial population distribution after calibration period

| Group | Susceptible | All HIV infected† | HIV infected and on ART†† |
| --- | --- | --- | --- |
| Children | 15,285,185 | 122,056 | 25,885 |
| Men | 8,353,903 | 342,481 | 109,713 |
| Women | 8,307,874 | 493,898 | 131,458 |
| Totals | 31,946,962 | 958,435 | 267,056 |

†Includes those HIV infected persons who are undetected (i.e. not tested), detected but not in care, and those in care and treatment programs (both on and off ART)

††Only those detected, in care and on ART

- 1. Entry and Maturation

Entry into the population was determined by the age-associated fertility rate for Uganda in 2008 and was distributed in relative proportion by risk group (p). Entry was assumed at birth but no sexual activity was assumed to occur until age 13. For any given compartment (not accounting for HIV infection or HIV-related death) entry, maturation and mortality were calculated as follows:

Newborns:

N_a=0,r_ (*t+1*) = Births*_r_* – ((1/D_a=0_) * N_a=0,r_ (*t*)) − μ_age_  (8)

Other age groups:

N_a,r_ (*t+1*) = ((1/D_a=0_)*N_a=0,r_ *(t)* )) – ((1/D_a_) * N_a,r_ *(t)* )) - μ_age_ (9)

Newborns acquire HIV through vertical transmission according to a probability that varies with the mother’s viral load. Mothers taking PMTCT meds are assumed to have a viral load two log strata lower than their actual viral load. (Ex. Women with a vl category of 5 will be attributed a vl category of 3 when determining the proportion of infected births.) Newborns are allocated equally across genders and are assigned into risk groups in the same proportions that adults are allocated. They are assigned CD4 and VL categories using the same allocation probabilities as adults who acquire new infection. Infected newborns inherit the resistance category of the mother.

**4 Outcomes**

The system of nonlinear differential equations were solved numerically to calculate the number of persons in each compartment over time. The following outcome measures were then derived.

- 1. Effectiveness

4.1.1 Total number of HIV infections was calculated at a given time t as:

Σ N _y>1_(t)

Where y refers to status variable (1=susceptible, >1 = HIV infected)

4.1.2 Prevalence at time t was calculated as:

Σ N _y>1_(t) / Σ N(t)

4.1.3 Number of new infections over time horizon T was calculated as:

New infections= ∫^T^ Σ λ_y>1_(t) * N_y=1_(t)dt (10)

4.1.4 Number of infections averted was calculated as:

# of new HIV infections (base case) - # of new HIV infections (intervention scenario)

- - 1. Life Years and QALYs

The total life years for each time cycle was calculated by summing the number of people over all compartments. To calculate QALYs, utility scores were stratified on CD4^+^ strata [13].

4.2 Costs

Costs that were accounted for included acute hospitalization costs secondary to AIDS-related clinical events, 1^st^ and 2^nd^ line ART drugs, monitoring costs (varied by different monitoring strategy considered), and routine outpatient care of HIV infected persons. Costs were discounted at 3%. The mean annual cost associated with each starting compartment was determined from the progression model and imported into the transmission model via the lookup tables. The total cost for each time cycle is the product of the number of people in each compartment multiplied by that compartment’s annual cost, summed over all compartments.

**Table S4. Key input parameters to simulation model**

| **Sexual risk characteristics** |  | **Value** | **Source** |
| --- | --- | --- | --- |
| **P_abs, k=2_** | Proportion of males who are abstinent | 5% | [14-16] |
| **Pr_l=2,k=2_** | Proportion of males who are monogamous | 31% | [16-18] |
| **Pr_l=3,k=2_** | Proportion of males in multiple, concurrent relationships (Class 3) | 56% | Assumption |
| **Pr_,l=2,k=2_** | Proportion of males in multiple, concurrent relationships (Class 4) | 8% | [19] |
| **P_abs, k=1_** | Proportion of females who are abstinent | 10% | [14, 20, 21] |
| **Pr_l=2,k=1_** | Proportion of females who are monogamous | 69% | [16, 17] |
| **Pr_l=3,k=1_** | Proportion of females in multiple, concurrent relationships | 17% | Assumption |
| **Pr_l=3,k=1_** | Proportion of females in multiple, concurrent relationships (Class 4) | 4% | [22] |
|  |  |  |  |
| **Sexual transmission** |  |  |  |
|  |  |  |  |
| **α^s^_k=2,K=1_** | Transmission risk per sex act (F🡪M) | 0.00042 | [23] |
| **α^s^_k=1,K=2_** | Transmission risk per sex act (M🡪F) | 0.00081 | [23] |
|  |  |  |  |
| **α^m^_v=0_** | Relative risk of transmission if VL category 0-2.5 log copies/ml | 0.16 | [24] |
| **α^m^_v=1_** | Relative risk of transmission if VL category 2.5-3.5 log copies/ml | 1.87 | [24] |
| **α^m^_v=2_** | Relative risk of transmission if VL category 3.5-4.5 log copies/ml | 6.54 | [24] |
| **α^m^_v=3_** | Relative risk of transmission if VL category 4.5-5.5 log copies/ml | 8.85 | [24] |
| **α^m^_v=4_** | Relative risk of transmission if VL category >5.5 log copies/ml | 9.03 | [24] |
| **ε1** | Degree of assortative mixing between age classes (0=assortative, 1=proportionate) | 0,25 | [4] |
| **ε2** | Degree of assortative mixing between sexual activity classes (0=assortative, 1=proportionate) | 0.20 | [4] |
| **d_l=1_** | Average duration (years) of stable, monogamous partnerships | 30.0 | Assumption |
| **d_l=2_** | Average duration (years) of partnership in activity group 2 | 1.0 | Assumption |
| **d_l=3_** | Average duration (years) of partnership in activity group 3 | 0.5 | Assumption |
| **r_l=1_** | Median number of concurrent partnerships for activity group 1 | 1.0 | [18] |
| **r_l=2_** | Median number of concurrent partnerships for activity group 2 | 3.0 | [18] |
| **r_l=3_** | Median number of concurrent partnerships for activity group 3 | 10.0 | [18] |
| **ε3** | For men over age 25, the proportion of partnerships which they would have formed with women in the same age group that are formed with women in the age group 10 years younger | 0.16 | [25] |
|  |  |  |  |
| **HIV risk behaviors and biological/behavioral modifiers of transmission** | |  |  |
| **P_STI_** | Prevalence of untreated STI | 6% | [26] |
| **P_circ_** | Probability of not being circumcised | 50% | [27, 28] |
| **P_alc_** | Initial proportion alcohol abuse problems, males | 20% | [29] |
|  | Initial proportion alcohol abuse problems, females | 10% | [29] |
| **P_condom_** | Prevalence of condom nonuse most or all of the time | 66% | [30] |
|  | Relative risk of alcohol/mental health/drug use on condom nonuse | 1.29 | [31, 32] |
|  | Relative risk of alcohol/mental health/drug use on ART nonadherence | 2.33 | [33-36] |
|  | Relative risk of alcohol/mental health/drug use on having an untreated STI | 1.72 | [37, 38] |
| **A^p^_condom_** | Relative risk reduction of HIV seroconversion when using condoms | 80% | [39] |
| **A^p^_PrEP_** | Relative risk reduction of HIV seroconversion when using PrEP | 44% | [40] |
| **A^p^_STI_** | Relative risk reduction of HIV seroconversion if treated STI | 40% | [41] |
| **A^p^_circ_** | Relative risk reduction of HIV seroconversion if circumcised | 59% | [42] |
|  |  |  |  |
| **HIV disease related** |  |  |  |
| **P_test_** | Probability/rate of not being tested for HIV | 84% | [43-45] |
| **P_LTC_** | Probability of linkage to care once HIV diagnosed | 68% | [46, 47] |
|  | Mean CD4 count (SD) for newly infected individuals | 705 (50) | [48] |
|  | Mean log viral load (SD) for newly infected females | 4.27 (1.15) | [49] |
|  | Mean log viral load (SD) for newly infected males | 4.99 (0.88) | [49] |
|  | CD4 count threshold for treatment initiation | 350 | [50] |
| **Padh** | Probability of nonadherence | 15% | [51, 52] |
|  |  |  |  |
| **Demographics** |  |  |  |
|  |  |  |  |
| **θ** | Fertility rate (range, depending on age) | 0.0291-0.3323 (29.1-332.3/1000 pop/year) | [53] |
|  |  |  |  |
| **Costs** |  |  |  |
|  | 1^st^ line ART monthly costs | $15.78 | Unpublished data^•^ ^•^ |
|  | 2^nd^ line ART monthly costs | $113.43 | Unpublished data^•^ ^•^ |
|  | Annual routine costs if in HIV care and treatment program | $287.28 | Unpublished data^•^ ^•^ |
|  | Annual inpatient hospitalization costs if patient has AIDS | $390.27 | Unpublished data^•^ ^•^ |
|  | HIV-1 viral load test | $70 | Unpublished data^•^ ^•^ |
|  | CD4+ count test | $11.20 | Unpublished data^•^ ^•^ |

**••**Values derived from communication with Wools-Kaloustian, K (Director of Field Research; Indiana Univeristy-AMPATH [Academic Model Providing Access to Healthcare])

1. **Calibration of HIV epidemic model**

We pre-specified three calibration criteria in order to evaluate whether the model’s predictions were compatible with observed results: HIV prevalence, people living with HIV, and AIDS-related mortality (Figure S3). We compared data from the most recent year available (2007), as well as time trends over the longest period of time (1997-2007) over which East Africa data were available for all three criteria (WHO 2008). We selected Uganda as the target country because it had particularly complete and accessible epidemiological data.

**Figure S3A-C. Calibration of East Africa HIV Simulation Model.**

Figure S3A. Model calibration of HIV prevalence over time. The dotted line shows model projections and the solid line shows estimated HIV prevalence from published literature (WHO 2008). The long dashed line shows a high estimate and the short dashed line shows a low estimate from the literature (WHO 2008).

Figure S3B. Model calibration of people living with HIV (PLWHIV) over time. The dotted line shows model projections and the solid line shows the estimated number of PLWHIV from published literature (WHO 2008). The long dashed line shows a high estimate and the short dashed line shows a low estimate from the literature (WHO 2008).

Figure S3C. Model calibration of HIV-related deaths over time. The dotted line shows model projections and the solid line shows estimated HIV-related deaths from published literature (WHO 2008). The long dashed line shows a high estimate and the short dashed line shows a low estimate from the literature (WHO 2008).

**3A**

**3B**

**3C**

**
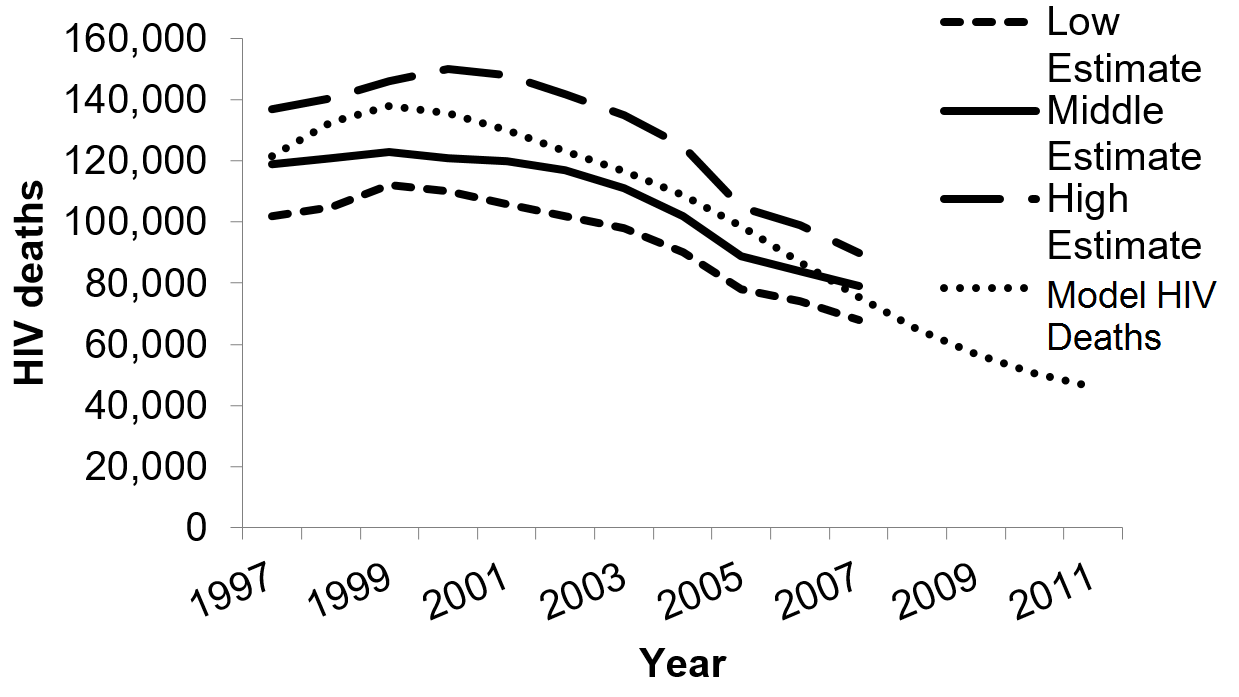
**

**6 Implementation of HIV prevention pathways and interventions**

HIV prevention interventions were hypothesized to affect one or more “pathways” modifying HIV sexual or needle based transmission (Figure S4). An intervention can affect one or more specific pathways that influence HIV transmission in one of five direct mechanisms in the model, a) applying a modifier to alpha (probability of transmission during sex or injection act between discordant pair); b) modifying/shifting proportions of relevant compartments; c) modifying implicit rates/probabilities d) direct modifier on HIV – related mortality rates; e) modify the clinical CD4 threshold for antiretroviral treatment initiation. The duration of any activated intervention and its effect in any specified model run was considered as binary (on/off) and remains so throughout the model time horizon.

**Figure S4 a-b.** HIV transmission “pathways” that are modified by prevention interventions a. Schematic of constructs in transmission simulation and pathways which impact HIV transmission. b. Mechanisms by which “pathways” interact with HIV transmission/acquisition risk and interventions influence “pathways”.

*Infected*

*Uninfected*

, High risk

Lower risk,

*Infected*

Lower risk,

*uninfected*

**Less risk per**

**event (horizontal**

**or vertical)**

**Lower risk**

**-**

**barrier**

**(condom)**

**Fewer**

**partners**

**Less IDU**

**Effective ART**

**Fewer risky**

**sex events**

**(horizontal)**

**Fewer risky**

**IDU events**

**(horizontal)**

**Lower risk**

**-non**

**-**

**barrier**

**(STD, prophyl ART),**

**)**

**Less**

**risky IDU**

**Increased HIV testing**

**Increased linkage to care** and treatment

**Increased adherence**

These pathways are not currently included in East Africa conceptualization of model

b. *Pathway mechanisms*

**Table S5. Pathway mechanisms**

| **Pathway** | **1**  **Lower risk -barrier :**  **Condom use** | **2**  **Lower risk -nonbarrier:**  **Prevalence of non-HIV STI’s** | **3**  **Lower risk non-barrier-**  **Other (PrEP, circumcision)** | **4**  **Number of sexual partners** | **5**  **Earlier ART: HIV testing** | **6**  **Earlier ART: HIV linkage** | **7**  **Effective ART: Adherence to ART** | **8**  **Effective ART:**  **Access to ART** |
| --- | --- | --- | --- | --- | --- | --- | --- | --- |
| **Intervention “mapped” to this pathway** | Impacts probability of consistent condom use | Impacts proportion of population with untreated STI | Impacts proportion of population using method | Impacts proportion of persons in multiple partnering compartment | Impacts probability of annual testing for HIV | Impacts probability of transition from HIV infected to HIV in care | Impacts probability of nonadherence among those on ART | CD4 threshold for initiating ART |
| **Model mechanism** | Sexual alpha modifier | Sexual alpha modifier | Sexual alpha modifier | Shift compartment proportions | Rate/Transition  modifier | Rate/Transition  Modifier | Progression modifier | Adjusted progression module reference table |

Every intervention had a point estimate (a relative risk or risk reduction) for its effect size () on each pathway, an upper and lower bound to represent the uncertainty of these estimates, and a target group (Table S1). If an intervention was thought not to affect a given pathway a null effect size was assumed and no bounds applied. One or more interventions could be “activated” during a simulation. Because the joint effectiveness of multiple biomedical interventions is unknown we assume, conservatively, that if more than one (n>1) intervention affect a given “pathway” and target group then the joint effectiveness is simply the maximum program effectiveness of the n interventions. However, if combinations of interventions affect independent pathways those effects are propagated without modification.

Effect sizes of interventions on “mapped” pathways were taken or derived from relevant literature sources. In cases where no relative risk (RR) was reported (e.g. odds ratios were used) review of the primary source and calculation of a relative risk (if applicable) was undertaken. If sufficient data was unavailable in primary source to calculate a RR then a conversion from odds ratio to risk ratio/relative risk was estimated using a published method (2). Where appropriate probabilities were converted to rates (r = - [ln (1 – P)] / t) and vice-versa (p = 1 – exp(-rt)).

The interventions that were considered in our analyses included :

| - Mass marketing HIV education campaigns [**MM**] |
| --- |
| - Structural (no-cost) condom distribution [**Co**] |
| - Enhanced PMTCT – (Option B for all)[**PMTCT**] |
| - Harm reduction for commercial sex workers [**CSW**] |
| - Voluntary medical male circumcision [**MMC**] |
| - Hazardous alcohol use screening and brief intervention [**SBIRT**] |
| - Expanded ART access- 2013 WHO guidelines [**WHO**] |
| - Oral pre-exposure prophylaxis for those HIV uninfected at high risk [**PrEP**] |
| - Test, link, treat for all; “treatment as prevention” [**TasP**] |

The transmission “pathways” modified, the effect sizes and costs of each of these interventions is outlined in Table S1.

Interventions acted on HIV transmission “pathways” in one or more of the following ways:

*1) Interventions which modify transmission probability per act (α)*

Recall from above the calculation for transmission probability is dependent upon partnership type and viral load of infected partner. Several of the considered interventions act along pathways that directly modify the transmission probability per act (green highlighted pathways in Figure 3b). To calculate the adjusted alpha under the intervention scenario, an alpha modifier related to the intervention is first calculated (α^m^_INT_).

α^m^_INT_= ((1-P_b_) – ((1-P_b_)*(1-τ)))*A^p^ + (1-((1-P_b_)-((1-P_b_)*(1-τ))))*1 (1)

Where P_b_= baseline probability; τ = effect size of intervention; A^p^= alpha multiplier of pathway

For example:

Assume condom distribution intervention activated which has an effect size of 12% (i.e. probability of using condoms consistently in target population is 12% greater), baseline probability of consistent condom use is 35% and the alpha modifier of condom use during sex is 0.2 (an 80% risk reduction in transmission).

α^m^_INT_= ((1-0.35) – ((1-0.35)*(1-0.12)))*0.2 + (1-((1-0.35)-((1-0.35)*(1-0.12))))*1 = 0.938

The final transmission probability then equals:

α_kKV_ = α_kK_ * α^m^_v_ * α^m^_INT_ (2)

*2) Interventions that modify compartment populations*

The shifting of proportions of specific compartments resulting from activation of interventions that act along these pathways is calculated as follows:

N_r_’=N_r_ * τ_INT_ (3)

where N_r_ refers to the total number within a specific compartment with risk characteristics r; τ is intervention effect size.

N_r_’ persons will move from specified risk strata usually to different risk strata depending on the specific intervention. For those interventions influencing the sexual partnering pathway , N_r_ refers to compartments where *l=3* (multiple partners). The resultant N_r_’ from the above equation are moved to relevant *l=2* compartments. Similarly for any interventions mapped to the IDU reduction pathway the N_r_ refers to all IDU compartments (j=2) and the calculated N_r_’ are moved to relevant non-IDU( j=1) compartments.

*3) Interventions that modify transition rates along spectrum of engagement in care*

The effect of interventions that act via pathways which modify  are calculated by applying the intervention effect sizes (τ_test_ or τ_link_) to the assumed probabilities of either HIV testing (P_test_) and or linkage to care (P_LTC_)as follows:

ν’ = (1-(1-P_test_*1-τ_test_ ))*(1-(1-P_LTC)_*(1-τ_link_)) (4)

For example:

Assume a testing intervention is implemented that has an effect size of 33% (i.e. increases annual probability of HIV testing by 33%; linkage to care probability unchanged). The baseline probability of HIV testing is 31% and the baseline probability of linkage to care is 74%.( ν= 0.23; 23%)

ν’ = (1- (1-0.31)*(1-0.33)) * (1- (1-0.74) * 1)) = ~ 0.40; 40%

*4) Interventions that modify adherence to ART*

Interventions that modify adherence to ART act by modifying the HIV-related mortality rate at a specified CD4 and VL combination. The probability of adherence under conditions when such an intervention is active is calculated by:

P_adh_’ = 1 - ((1-P_adh_) * (1-τ_int_)) (5)

A modifier that defines the relative risk of mortality at a given level of adherence ([adh_y_] see table 5) is then applied to the HIV-associated mortality rate (’_cd,v_). If the value of P_adh_’ is between two values a linear interpolation is performed.

μ_INT_= μ_cd,v_ * Madh_y_ (6)

For example:

Assume an adherence intervention is implemented that has an effect size of 20% (i.e. probability of adherence is increased by 20%, or conversely non-adherence is decreased by 20%). The baseline probability of adherence is 63%.

P_adh_’ = 1- ((1-0.63)*(1-0.20)) = 0.70 ~ 70%

From table S2 Madh_y_ =0.993 then μ_INT_ = μ_cd,v_ * 0.993

5) *Interventions that modify when to initiate antiretroviral therapy*

These interventions (e.g. implementation of 2013 WHO guidelines) act to increase the number of persons eligible to receive ART based on clinical parameters (CD4 count). The effectiveness of ART is assumed to be independent of CD4.

**Table S6**. *Portfolio of HIV prevention interventions included in computer simulation and their operating characteristics.* HIV transmission pathways as described in Figure S1a and b. Numbers in cells represent relative risks or costs in 2012 USD (plausible ranges).

|  |  | **Pathway** | | | | | | | |  | |
| --- | --- | --- | --- | --- | --- | --- | --- | --- | --- | --- | --- |
|  | **Target pop** | **1** | **2** | **3** | **4** | **5** | **6** | **7** | **8** | **Cost** | **Refs** |
| **Intervention** | | | | | | | | | | | |
| Mass marketing [**MM**] | All | 1.09  (1.01-1.33) |  |  |  | 1.07  (1.01-1.20) |  |  |  | $1  ($0.50-$1.50) | (3–8) |
| Condom distribution [**Co**] | All | 1.23  (1.06-1.31) | 0.69  (0.52-0.91) |  |  |  |  |  |  | $5  ($1-$10) | (9,10) |
| Enhanced PMTCT [**PMTCT**] | All women |  |  | ***a*** |  | 4.0  (2.5-8.0) |  |  |  | $65  ($32-$97) | (11–13) |
| Harm reduction for CSW [**CSW**] | Female CSW | 3.40  (1.3-4.0) | 0.31  (0.01-0.51) |  |  |  |  |  |  | $290  ($145-$435) | (14–16) |
| Voluntary medical male circumcision [**MMC**] | HIV - men |  |  | 2.4***^b^***  (1.5-4.0) |  |  |  |  |  | $58  ($46-$69) | (17–21) |
| Hazardous alcohol use screening and brief intervention [**Alc**] | All | ***c*** | ***c*** |  |  |  |  | ***c*** |  | $5 | (22,23) |
| Expanded ART access- 2013 WHO guidelines [**WHO**] | All HIV + |  |  |  |  |  |  |  | ≤500 cells/mm^3^ | -- | (24) |
| Oral pre-exposure prophylaxis [**PrEP**] | All HIV – at high risk**^†^** of HIV |  |  | 2.0***^d^***  (1.1-10) |  |  |  |  |  | $165  ($80-$250) | (25,26)(27–31) |
| Test, link, treat for all [**TasP**] | All |  |  |  |  | 1.70  (1.5-2.0) | 2.0  (1-4) |  | All | $39  ($23-$67) | (32,33) |

**^a^** PMTCT intervention assumes that WHO option B will be utilized by 80% of pregnant women. Use of ART in pregnacy is associated with a 97% relative risk reduction in transmission to infant.

**^b^** Circumcision is associated with a 40% relative risk reduction for male acquisition of HIV in a F/M serodiscordant relationship.

**^c^** Alc intervention reduces proportion of hazardous alcohol misusers in the adult population by 45% (20%-70%). Hazardous alcohol misuse is associated with a increased relative risk of condom mis/nonsue (1.29); prevalence of non-HIV STI’s (1.72); and non-adherence to ART (2.33).

**^d^** Use of PrEP is associated with a relative risk reduction of 70% on HIV acquisition in serodiscordant couples.

† High-risk refers to persons who engage in multiple, concurrent partnerships or are commercial sex workers or their clients.**References**

1. Braithwaite, R.S., et al., *Estimating the proportion of patients infected with HIV who will die of comorbid diseases.* The American journal of medicine, 2005. **118**(8): p. 890-8.

2. Braithwaite, R.S., et al., *Alternative antiretroviral monitoring strategies for HIV-infected patients in east Africa: opportunities to save more lives?* J Int AIDS Soc, 2011. **14**: p. 38.

3. Garnett, G.P. and R.M. Anderson, *Factors controlling the spread of HIV in heterosexual communities in developing countries: patterns of mixing between different age and sexual activity classes.* Philos Trans R Soc Lond B Biol Sci, 1993. **342**(1300): p. 137-59.

4. Garnett GP, A.R., *Balancing sexual partnership in an age and activity stratified model of HIV transmission in heterosexual populations.* Journal of Mathematics Applied in Medicine & Biology, 1994. **11**: p. 161-192.

5. Kahn, J.O. and B.D. Walker, *Acute human immunodeficiency virus type 1 infection.* The New England journal of medicine, 1998. **339**(1): p. 33-9.

6. Morgan, D., et al., *HIV-1 RNA levels in an African population-based cohort and their relation to CD4 lymphocyte counts and World Health Organization clinical staging.* J Acquir Immune Defic Syndr, 1999. **22**(2): p. 167-73.

7. Saathoff, E., et al., *Viral and host factors associated with the HIV-1 viral load setpoint in adults from Mbeya Region, Tanzania.* J Acquir Immune Defic Syndr, 2010. **54**(3): p. 324-30.

8. Williams, B.G., et al., *HIV infection, antiretroviral therapy, and CD4+ cell count distributions in African populations.* J Infect Dis, 2006. **194**(10): p. 1450-8.

9. Surveillance, U.W.W.G.o.G.H.A.a.S., *UNAIDS/WHO Epidemiological Fact Sheets on HIV and AIDS, 2008 Uganda*. 2009, UNAIDS/WHO: Geneva.

10. United Nations, D.o.E.a.S.A., Population Division, *World population prospects: the 2008 revision*. 2008.

11. Kamali, A., et al., *Seven-year trends in HIV-1 infection rates, and changes in sexual behaviour, among adults in rural Uganda.* AIDS, 2000. **14**(4): p. 427-34.

12. *Kenya AIDS indicator survey*. 2007.

13. Freedberg, K.A., et al., *The cost-effectiveness of preventing AIDS-related opportunistic infections.* Jama, 1998. **279**(2): p. 130-6.

14. Kapiga, S.H., et al., *The epidemiology of HIV-1 infection in northern Tanzania: results from a community-based study.* AIDS care, 2006. **18**(4): p. 379-87.

15. Mbizvo, M.T., et al., *HIV seroincidence and correlates of seroconversion in a cohort of male factory workers in Harare, Zimbabwe.* AIDS, 1996. **10**(8): p. 895-901.

16. Quigley, M., et al., *Sexual behaviour patterns and other risk factors for HIV infection in rural Tanzania: a case-control study.* AIDS, 1997. **11**(2): p. 237-48.

17. Mishra, V. and S. Bignami-Van Assche, *Concurrent Sexual Partnerships and HIV Infection: Evidence from National Population-Based Surveys. DHS Working Papers No. 62.*, in *Calverton, Maryland: Macro International Inc.* 2009.

18. Volle, J., et al., *A baseline survey of multiple and concurrent sexual partnerships among Basotho men in Lesotho*, C-Change/AED, Editor. 2009: Washington, DC.

19. Mmbaga, E.J., et al., *Sexually transmitted infections knowledge and its impact in the practice of risky sexual behaviours and HIV serostatus: results from rural Kilimanjaro, Tanzania.* Sexually Transmitted Infections, 2008. **84**(3): p. 224-226.

20. Ao, T.T., et al., *Human immunodeficiency virus type 1 among bar and hotel workers in northern Tanzania: the role of alcohol, sexual behavior, and herpes simplex virus type 2.* Sexually Transmitted Diseases, 2006. **33**(3): p. 163-169.

21. Kapiga, S.H., et al., *HIV-1 epidemic among female bar and hotel workers in northern Tanzania: risk factors and opportunities for prevention.* Journal of acquired immune deficiency syndromes, 2002. **29**(4): p. 409-17.

22. Vandepitte, J., et al., *Estimates of the number of female sex workers in different regions of the world.* Sexually transmitted infections, 2006. **82 Suppl 3**: p. iii18-25.

23. Boily, M.-C., et al., *Heterosexual risk of HIV-1 infection per sexual act: systematic review and meta-analysis of observational studies.* The Lancet Infectious Diseases, 2009. **9**(2): p. 118-129.

24. Attia, S., et al., *Sexual transmission of HIV according to viral load and antiretroviral therapy: systematic review and meta-analysis.* AIDS, 2009. **23**(11): p. 1397-404.

25. Wawer, M.J., et al., *Rates of HIV-1 transmission per coital act, by stage of HIV-1 infection, in Rakai, Uganda.* The Journal of infectious diseases, 2005. **191**(9): p. 1403-9.

26. World Health Organization, *Global Prevalence and Incidence of Selected Curable Sexually Transmitted Infections Overview and Estimates*. 2001: Geneva.

27. Lissouba, P., et al., *Adult male circumcision as an intervention against HIV: an operational study of uptake in a South African community (ANRS 12126).* BMC infectious diseases, 2011. **11**: p. 253.

28. World Health Organization and UNAIDS, *Male circumcision: global trends and determinants of prevalence, safety and acceptability*. 2007.

29. Nacada Authority for a Drug Free Nation, *Alcohol Abuse and HIV Infection in Nairobi - Survey.* 2010.

30. Westercamp, N., et al., *Determinants of consistent condom use vary by partner type among young men in Kisumu, Kenya: a multi-level data analysis.* AIDS and behavior, 2010. **14**(4): p. 949-959.

31. Weiser, S.D., et al., *Food insufficiency is associated with high-risk sexual behavior among women in Botswana and Swaziland.* PLoS medicine, 2007. **4**(10): p. 1589-97; discussion 1598.

32. Weiser, S.D., et al., *A population-based study on alcohol and high-risk sexual behaviors in Botswana.* PLoS medicine, 2006. **3**(10): p. e392.

33. Nduna, M., et al., *Associations between depressive symptoms, sexual behaviour and relationship characteristics: a prospective cohort study of young women and men in the Eastern Cape, South Africa.* Journal of the International AIDS Society, 2010. **13**: p. 44.

34. Nakimuli-Mpungu, E., et al., *Psychological distress and adherence to highly active anti-retroviral therapy (HAART) in Uganda: a pilot study.* African health sciences, 2009. **9 Suppl 1**: p. S2-7.

35. Tadios, Y. and G. Davey, *Antiretroviral treatment adherence and its correlates in Addis Ababa, Ethiopia.* Ethiopian Medical Journal, 2006. **44**(3): p. 237-244.

36. Byakika-Tusiime, J., et al., *Longitudinal antiretroviral adherence in HIV+ Ugandan parents and their children initiating HAART in the MTCT-Plus family treatment model: role of depression in declining adherence over time.* AIDS and behavior, 2009. **13 Suppl 1**: p. 82-91.

37. Fisher, J.C., et al., *Patterns of alcohol use, problem drinking, and HIV infection among high-risk African women.* Sexually transmitted diseases, 2008. **35**(6): p. 537-44.

38. Alem, A., et al., *Unprotected sex, sexually transmitted infections and problem drinking among female sex workers in Ethiopia.* Ethiopian Journal of Health Development, 2006. **20**(2): p. 93-98.

39. Weller SC, D.-B.K., *Condom effectiveness in reducing heterosexual HIV transmission.* Cochrane Database of Systematic Reviews, 2002(1).

40. Grant RM, L.J., Anderson PL, et al., *Preexposure chemoprophylaxis for HIV prevention in men who have sex with men.* New England Journal of Medicine, 2010. **363**(27): p. 2587-2599.

41. Grosskurth H, M.F., Todd J, et al, *Impact of improved treatment of sexually transmitted diseases on HIV infection in rural Tanzania: randomised controlled trial.* Lancet, 1995. **346**: p. 530-536.

42. Gray, R.H., et al., *Probability of HIV-1 transmission per coital act in monogamous, heterosexual, HIV-1-discordant couples in Rakai, Uganda.* Lancet, 2001. **357**(9263): p. 1149-53.

43. Irungu, T.K., et al., *HIV voluntary counselling and testing in Nakuru, Kenya: findings from a community survey.* HIV medicine, 2008. **9**(2): p. 111-117.

44. Mossdorf, E., et al., *Impact of a national HIV voluntary counselling and testing (VCT) campaign on VCT in a rural hospital in Tanzania.* Tropical medicine & international health : TM & IH, 2010. **15**(5): p. 567-573.

45. *Efficacy of voluntary HIV-1 counselling and testing in individuals and couples in Kenya, Tanzania, and Trinidad: a randomised trial. The Voluntary HIV-1 Counseling and Testing Efficacy Study Group.* Lancet, 2000. **356**(9224): p. 103-12.

46. Nsigaye, R., et al., *From HIV diagnosis to treatment: evaluation of a referral system to promote and monitor access to antiretroviral therapy in rural Tanzania.* Journal of the International AIDS Society, 2009. **12**(1): p. 31.

47. Rosen, S. and M.P. Fox, *Retention in HIV care between testing and treatment in sub-Saharan Africa: a systematic review.* PLoS medicine, 2011. **8**(7): p. e1001056.

48. Morgan, D., et al., *HIV-1 RNA levels in an African population-based cohort and their relation to CD4 lymphocyte counts and World Health Organization clinical staging.* Journal of acquired immune deficiency syndromes, 1999. **22**(2): p. 167-73.

49. Saathoff, E., et al., *Viral and host factors associated with the HIV-1 viral load setpoint in adults from Mbeya Region, Tanzania.* Journal of acquired immune deficiency syndromes, 2010. **54**(3): p. 324-30.

50. AMPATH. *The Academic Model Providing Access to Healthcare*. Available from: <http://www.ampathkenya.org/>.

51. Bajunirwe, F., et al., *Adherence and treatment response among HIV-1-infected adults receiving antiretroviral therapy in a rural government hospital in Southwestern Uganda.* Journal of the International Association of Physicians in AIDS Care, 2009. **8**(2): p. 139-47.

52. Johannessen, A., et al., *Virological efficacy and emergence of drug resistance in adults on antiretroviral treatment in rural Tanzania.* BMC infectious diseases, 2009. **9**: p. 108.

53. Population Division of the Department of Economic and Social Affairs of the United Nations Secretariat, *World Population Prospects: The 2008 Revision.*
